# The familial aggregation and co-aggregation of drug use disorder and alcohol use disorder in siblings of affected individuals born 1950–1990: A birth cohort exposed to rising rates of drug use disorder

**DOI:** 10.1017/S0033291725000327

**Published:** 2025-03-05

**Authors:** Kenneth S. Kendler, Linda Abrahamsson, Jan Sundquist, Kristina Sundquist

**Affiliations:** 1 Virginia Institute for Psychiatric and Behavioral Genetics, Virginia Commonwealth University, Richmond, VA, USA; 2 Department of Psychiatry, Virginia Commonwealth University, Richmond, VA, USA; 3 Center for Primary Health Care Research, Lund University, Malmö, Sweden

## Abstract

**Background:**

We seek to clarify how changes in the prevalence of drug use disorder (DUD) in Sweden in the 1950–1990 birth cohort impact the aggregation and co-aggregation in siblings of DUD and alcohol use disorder (AUD).

**Methods:**

We examined risk for DUD and AUD in siblings of 102,624 DUD cases and matched control probands and 123,837 AUD case and matched control probands identified using Swedish registries. Flexible parametric survival models assessed the difference in disorder risk in siblings of case versus control probands.

**Results:**

Over birthyears 1950–1990, rates of DUD increased substantially in the Swedish population. In siblings of DUD cases versus controls, the risk for DUD increased dramatically starting in birthyear 1965 while their risk for AUD fell moderately. A similar, but less pronounced pattern, was seen in the siblings of AUD versus control probands. These differences were much larger in male than in female siblings.

**Conclusions:**

The factors that drove upward population rates of DUD in Sweden (e.g. increased availability, reduced stigma) produced much stronger effects in high-risk subjects (siblings of DUD and AUD probands) than in normal risk groups (siblings of controls), thereby increasing familial aggregation of DUD. However, parallel declines in AUD rates in high-risk versus normal-risk siblings were observed, likely due to ‘competitive effects’ reducing coaggregation of DUD and AUD. Results of genetic studies of substance use disorders can be substantially impacted by changes in availability and stigma of psychoactive substance use and indirectly by ‘competition’ as predicted by behavioral economic models, between abusable substances.

Prior studies of gene-environmental interaction in the etiology of substance use and substance use disorders (SUDs) have focused largely on measures of social constraint (e.g. high versus low parental monitoring, rural versus urban environments, low versus high peer deviance, strong versus low religious affiliation) and show, with considerable consistency, that heritability rises as constraints decrease (Chartier, Karriker-Jaffe, Cummings, & Kendler, 2017; Kendler et al., 2012; Koopmans, Slutske, van Baal, & Boomsma, 1999; Prom-Wormley, Ebejer, Dick, & Bowers, 2017). A few studies have also examined self-report measures of substance availability showing that high availability is associated with greater heritability of use (Kendler, Gardner, & Dick, 2011).

To further understand how substance availability might impact not only the aggregation of SUDs in relatives, but also the co-aggregation of different forms of SUD, in this paper, we take advantage of a natural experiment in Sweden for individuals born from 1950 to 1990 who experienced, over their lives, a substantial increase in prevalence of illicit drug use disorder (DUD). Specifically, we determine how such changes in prevalence—likely driven by changes in the availability and associated stigma of illicit substances—impact the patterns of aggregation and coaggregation of AUD and DUD in siblings.

We will do this by identifying case siblings with DUD and AUD and matched controls, and quantify, in these sibships, changes in the patterns of the aggregation and coaggregation of DUD and AUD experienced up through 2018 for those born from 1950 to 1990. We predict that in the part of our birth cohort that experienced rising rates of DUD in the Swedish population, we will see increases in the risks of DUD in the siblings of both DUD and AUD probands. Of particular interest will be whether, as predicted by behavioral economic competitive models, the rates of AUD will decline in the siblings of both the DUD and AUD probands during the relevant years. Because of the expected differences in prevalences of DUD and AUD in men versus women, we will also examine our sibling results as a function of the sex of the affected proband sibling and of the co-sibling. The broader goals of these analyses are to clarify how population shifts in availability and acceptability of different substances of abuse can impact the aggregation and co-aggregation within families for relevant forms of SUDs.

## Methods

Information for this study was collected from nationwide Swedish registers (Appendix A: Table 1). Each person’s unique identification number, replaced with a serial number for confidentiality, was used for registry linkages. Ethical approval was secured from the regional ethical review board in Lund, Sweden. Diagnoses of DUD and AUD were searched for using the Swedish Hospital Discharge Register, Outpatient Care Register, almost nationwide primary care data, the Swedish Prescribed Drug Register, the Swedish Cause of Death Register, the Swedish Criminal Register, and the Swedish Suspicion Register. For details, see Appendix A: Table 2.

We found n = 3,867,442 individuals born in Sweden between 1950 and 1994, having Swedish-born parents, not dying or migrating before age 24 or prior to 1969. These cut-off values were used to ensure we had adequate follow-up time to retrieve relevant registry diagnoses. Inclusion of individuals born between 1991 and 1994 were made to stabilize parametric model results (by minimizing extrapolation in the tails of the upper birthyear distribution, affected by the late increase in DUD incidence). The source population individuals were grouped into 2,072,471 unique parental pairs. Out of these, we were interested in the 1,257,132 unique parental pairs where we could find a sibship size of at least two full-siblings. The sibships were made up of 3,052,103 individuals. These individuals, with lifetime DUD and AUD prevalences of 4.1% and 4.9%, respectively, were used for finding case and control probands. Out of the 1,257,132 sibships, we found 111,181 (133,121) sibships where at least one sibling had a lifetime diagnosis of DUD (AUD). This gave us a total of 288,694 (355,745) individuals included within the DUD (AUD) sibships, with 125,451 (149,901) individuals affected with DUD (AUD), here called the DUD (AUD) case probands. For each proband, we matched, by sex, and birth year, one control proband. Further we applied incidence density sampling to ensure that the matched control proband had follow-up at least until the same age as the DUD (AUD) diagnosis of the case proband, and that the control proband had not been diagnosed with DUD (AUD) at that time point.

As there was an overlap (n = 47,821) between some of the DUD and AUD probands (and some co-siblings are diagnosed with both disorders), we assigned the comorbid cases the most dominant by the two disorders, according to an algorithm accounting for the number and timing of each diagnosis (Appendix A: Table 3). This simplification was important in the interpretation of our findings so that each individual was only represented once in our analyses. Before running the algorithm, both DUD and AUD registrations were cleaned so that two diagnoses of the same type occurring within 90 days of each other were counted as one (for details see Appendix A: Table 3).

For visualization of lifetime prevalences of DUD and AUD amongst all possible probands (n = 3,052,103) as a function of year of birth of proband, the prevalences were corrected to account for individuals born later having a shorter follow-up time. The correction was made such that individuals with follow-up time of 45 years or older were counted as 1 individual, individuals with shorter follow-up time was counted as a fraction of an individual, with the fraction following a straight line from 0 (at follow-up time 18 years) to 1 (at follow-up time 45 years).

In full-siblings to case and control probands, we analyze impact of DUD and AUD case–control proband status on time to first diagnosis of DUD and AUD in co-siblings, using a multivariable flexible parametric survival model (FPM) (Royston & Lambert, 2011; Royston & Parmar, 2002), through the function stpm2 in the R package rstpm2(Clements, 2019). We specifically utilized an additive hazards (AH) link function, available in the stpm2 function. The time scale used was age of co-sibling (from age of ten) and individuals were followed until time point of diagnosis, study end of 2018-12-31, death or emigration, whichever occurred first. Four different types of main models were performed: risk of DUD in co-siblings of DUD case probands, risk of AUD in co-siblings of DUD case probands, risk of DUD in co-siblings of AUD case probands, and risk of AUD in co-siblings of AUD case probands. In FPMs the baseline hazard is modelled, on the contrary to what is done in Cox proportional hazards models. For the modelling we make use of a natural spline (Perperoglou, Sauerbrei, Abrahamowicz, & Schmid, 2019) with five degrees of freedom. The parametric baseline hazard modelling has the attractive property that it enables model post-estimations (in stpm2 via the predict function) (Clements, 2019). This means that we may transform our results from, for example, the hazard scale to the survival scale. Stratification by the matched case–control proband pair, as one could have used in a Cox model, is not enabled in FPMs, but as we have performed matching on the exposure proband, and not on the outcome, and have information on the variables used to create the matching, we perform variable adjustment (Batyrbekova et al., 2022).

We apply a proband correction procedure proposed by Weinberg (Stark & Seneta, 2013) and utilized by Rhee, Abrahamsson, Sundquist, Sundquist and Kendler (2024)). As in Rhee et al., when applying the correction, for both case and control proband data, we allow an affected individual, to be counted as proband, once for each co-sibling, and to be counted as co-sibling, once for each affected sibling.

In the FPM, taking risk of DUD in co-siblings of DUD case probands, as an example, the effect that we are primarily interested in quantifying is the interaction effect between DUD case–control proband status and year of birth in proband. Proband year of birth is modeled with linear and quadratic main and interaction terms. A main effect of DUD case–control proband status is also included in the model. Variable adjustment is made for co-sibling birthyear, sex, and proband age at onset (AAO). The latter adjustment is made as AAO can index genetic risk for DUD (Kendler, Ohlsson, Bacanu, Sundquist, & Sundquist, 2023), and mean AAO is changing with proband year of birth. Co-sibling year of birth is modelled with linear, quadratic, and cubic main effects, to avoid residual bias. Co-sibling sex and birthyear (the linear term), as well as proband AAO, are modeled flexibly as time-varying effects by the inclusion of natural splines. For further details of these analyses, see Appendix A: Table 5a–5f. To estimate sex effects, we apply the same type of models, stratified by firstly proband sex, and secondly by sex in co-sibling.

In two sensitivity analyses, the same analyses were performed; firstly the unstratified analyses only, excluding any diagnosis from the primary health care data, and secondly unstratified and stratified by proband and co-sibling sex, without making use of the algorithm determining the most dominant disorder in comorbid probands and co-siblings.

Levels of significance of 0.05, 0.01, 0.001 and 0.0001 are marked in tables. Data analysis was conducted from March 7 to July 11, 2024 using R, version 4.3.1 (Team, 2023) (Appendix A: Table 4) and SAS, version 9.4(Inc., 2012).

## Results

### Population findings

Figures 1a, 1b, 1c depict the general sibling population prevalences of AUD and DUD by year of birth from 1950 to 1990 in the entire population, and then separately in males and females. All cases are divided into five mutually exclusive diagnostic groups: i) AUD only, ii) DUD only, iii) both AUD and DUD with AUD dominant, iv) both AUD and DUD with DUD dominant, and v) both AUD and DUD with neither dominant. In the entire population (figure 1a), we see, from birth year 1950 to 1970, a gradual decline in rates of AUD only with most other categories being relatively stable. From 1970 to 1990, the picture changes substantially with first a slow and then, after 1980, a rapid rise in rates of DUD only and AUD and DUD with DUD dominant. In these birth years, we first see a slightly decline in AUD only and then stability in those rates.

**Figure 1a.**
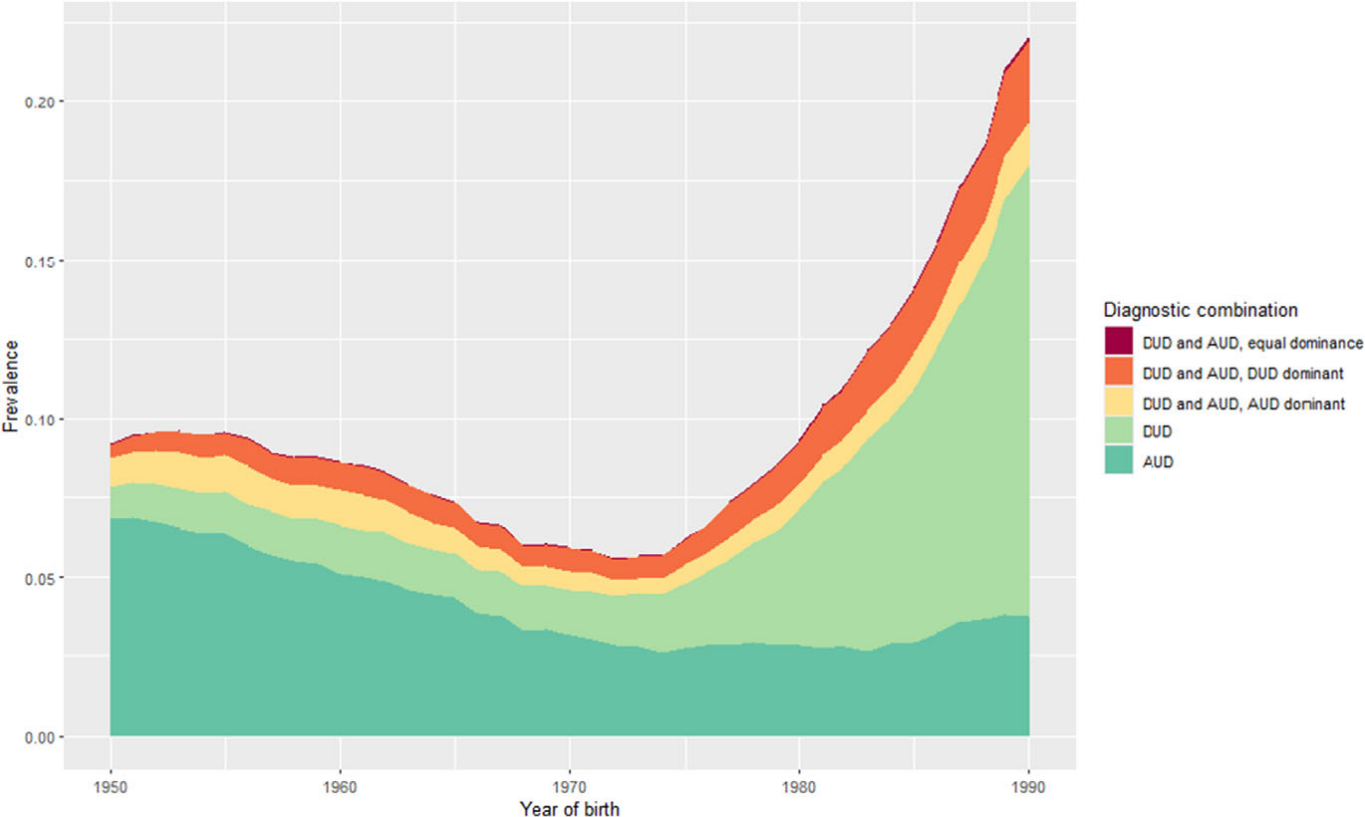
Lifetime prevalence in probands, with different diagnostic combinations and born in different years.

**Figure 1b and c.**
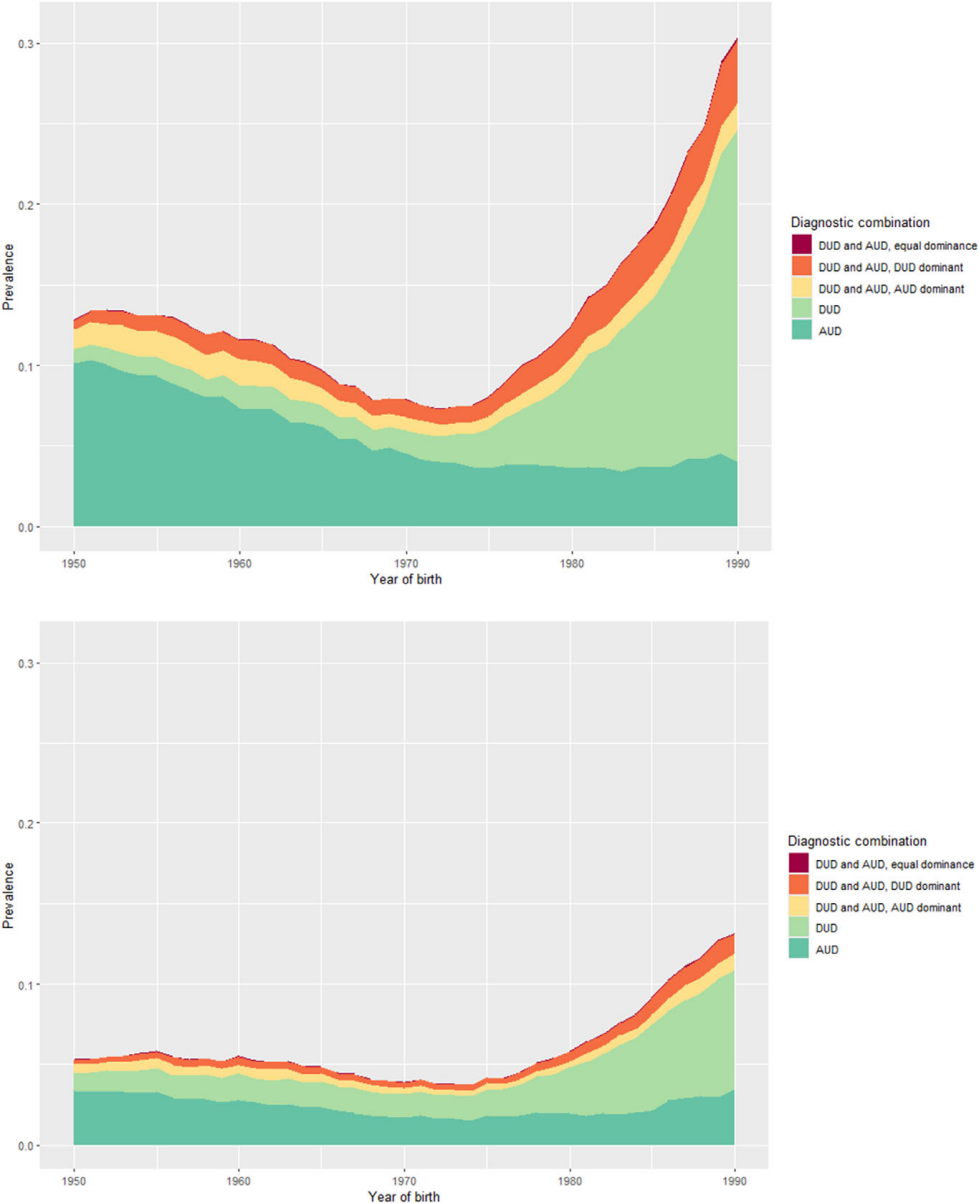
Lifetime prevalences in probands, with different diagnostic combinations and born in different years—stratified by proband sex, male followed by female.

The pattern in men (figure 1b) differs considerably with a gradual decline across the entire time period in rates of AUD only especially before 1975, the same year that the rates of DUD (and DUD and AUD with DUD dominant) start to rise dramatically. In women (figure 1c), the post 1975 rise in rates of DUD is less dramatic than in men as is the fall in rates of AUD.

### Sibling analysis descriptive results

Table 1 presents the main descriptive results of the sibling analyses. We identified 102,624 DUD cases (67% male) and 123,837 AUD proband cases (72% male) each with a matched control. In both probands and affected siblings, the age at onset was earlier in the DUD than AUD cases. A large proportion of both cases and controls had only one co-sibling for analysis. The morbid risk for DUD was 19.4% in the siblings of the DUD cases and 5.4% in the siblings of controls. The morbid risk for AUD was 13.5% in the siblings of AUD cases and 5.5% in the siblings of controls. We also saw increased co-aggregation of AUD in the siblings of DUD versus control proband and DUD in the siblings of AUD versus control probands.

**Table 1:** Descriptives of the total cohort. AUD and DUD diagnoses are based on applying a hierarchy to find the most dominant disorder

	DUD Case Proband	DUD Control Proband	AUD Case Proband	AUD Control Proband	
Number	102,624	102,624	123,837	123,837	
% Female	33.1	33.1	28.0	28.0	
Birth year, mean (sd)	1977.8 (12.4)	1977.8 (12.4)	1966.1 (11.5)	1966.1 (11.5)	
DUD prevalence, %^a^	100	2.4	0	2.3	
AUD prevalence, %^a^	0	3.2	100	3.1	
Age at onset of DUD, mean (sd)	28.9 (11.4)	28.0 (9.4)	NA	33.7 (13.4)	
Age at onset of AUD, mean (sd)	NA	33.4 (13.3)	36.1 (13.6)	45.5 (12.0)	
Number of siblings, median (IQR)	1 (1–2)	1 (1–2)	1 (1–2)	1 (1–2)	
Siblings to probands					General population
Number	166,721	164,911	218,140	210,196	3,867,442
% Female	48.2	48.2	48.7	48.3	48.5
Birth year, mean (sd)	1976.2 (12.6)	1976.4 (12.5)	1966.0 (11.3)	1966.2 (11.3)	1971.6 (13.1)
DUD prevalence, %	13.3	3.7	5.3	2.5	3.7
AUD prevalence, %	6.9	3.4	11.6	4.7	4.3
Morbid risk of DUD, %^a^	19.4	5.4	6.2	2.9	4.8
Morbid risk of AUD, %^a^	10.1	4.9	13.5	5.5	5.7
Age at onset of DUD, mean (sd)	25.9 (9.7)	26.4 (9.9)	33.0 (12.5)	33.6 (12.9)	28.7 (11.5)
Age at onset of AUD, mean (sd)	31.4 (12.6)	33.5 (13.2)	35.8 (13.6)	38.2 (14.1)	36.5 (13.9)
Age at follow-up, mean (sd)	41.5 (12.4)	41.5 (12.4)	51.1 (11.7)	51.2 (11.8)	45.9 (13.1)

a The morbid risk represents the lifetime prevalence, with the difference that the “at risk-set” in the denominator is age-corrected so that any individual under age 18 counts 0, ages 18–44 then count 0.5 and 45 and older count 1.

### Main sibling analyses

The key results are presented in figures 2a, 2b and 2c for, respectively, all co-siblings of all probands, separately for all co-siblings of male and female probands and separately for male and female co-siblings of all probands. All analyses present the differences in risk for DUD and AUD in co-siblings of case versus matched control probands up to age 40. That is, increasing values on the y-axis reflect increasing differences in risk of illness in siblings of the case probands compared to siblings of the control probands.

**Figure 2a.**
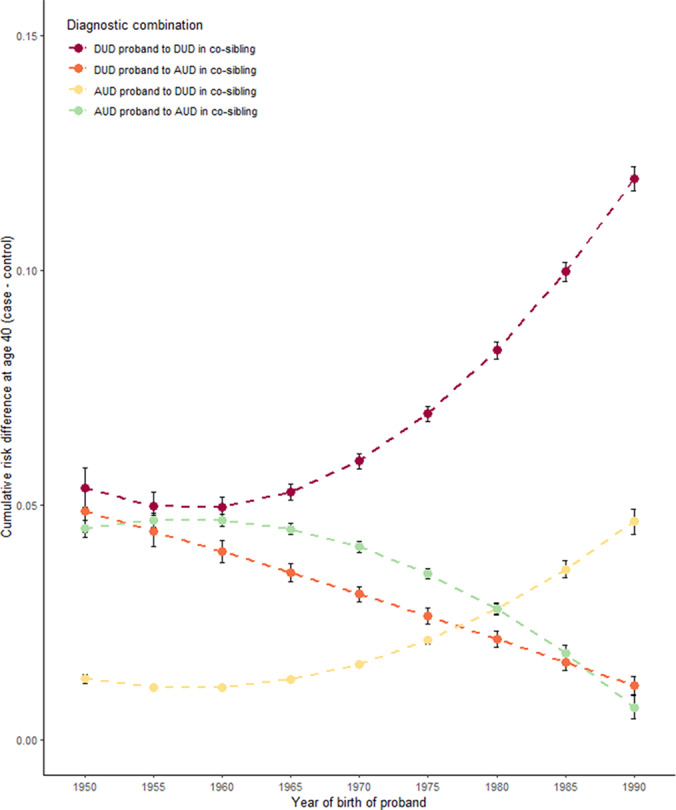
Predicted mean cumulative risk (=1-survival) differences (case co-siblings—control co-siblings) at age 40 in co-siblings.

**Figure 2b.**
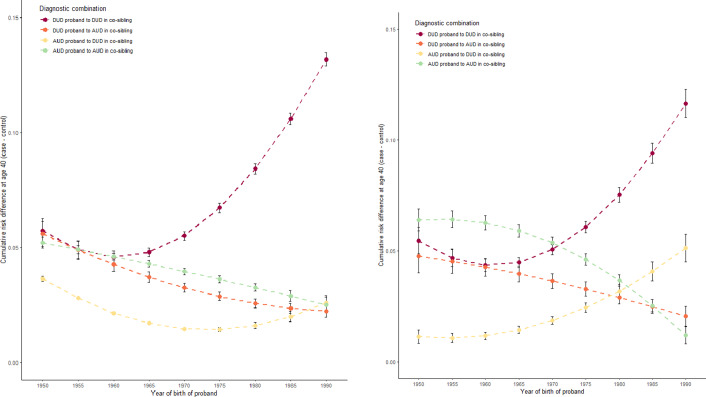
Predicted mean cumulative risk (=1-survival) differences (case co-siblings—control co-siblings) at age 40 in co-siblings. The figure to the left is stratified for proband sex being male, to the right female.

**Figure 2c.**
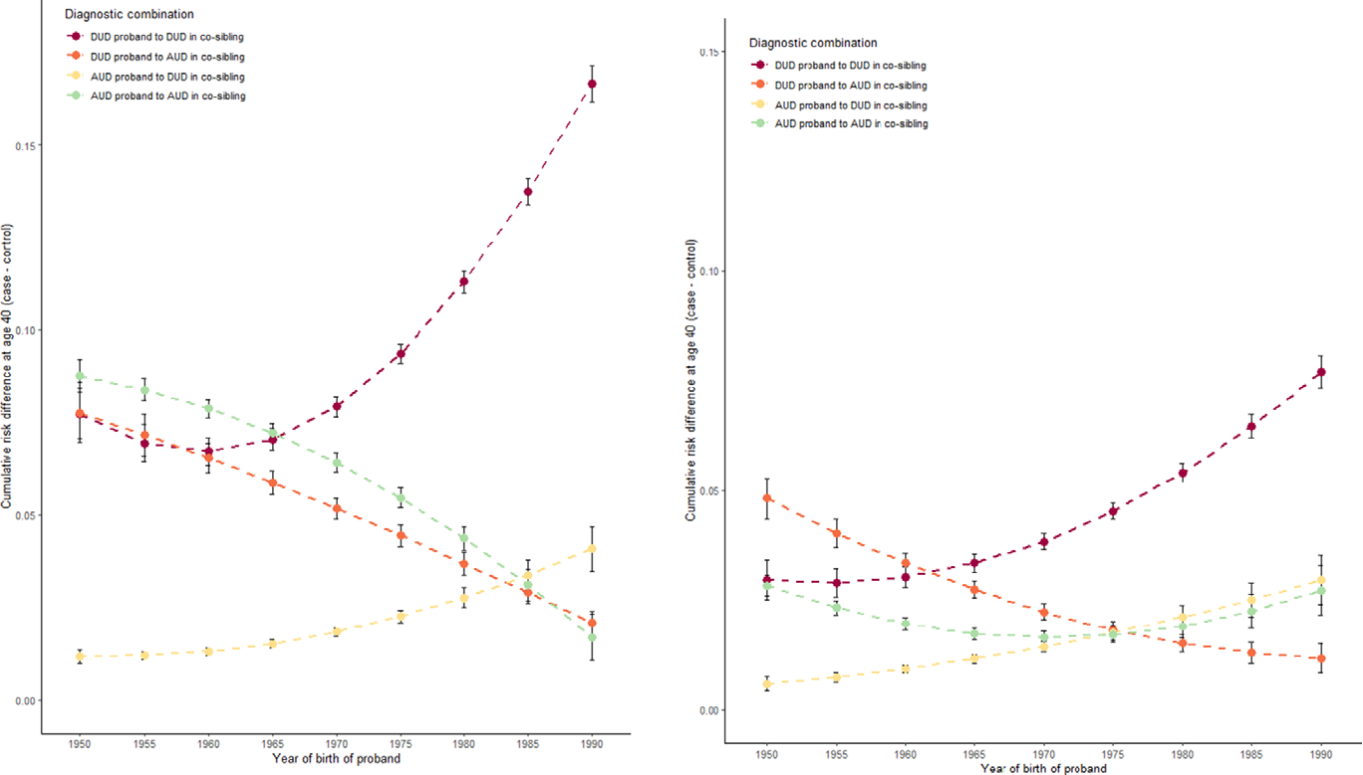
Predicted mean cumulative risk (=1-survival) differences (case co-siblings—control co-siblings) at age 40 in co-siblings. The figure to the left is stratified for co-sibling sex being male, and to the right is female.

Furthermore, each figure presents four lines with 95% CIs representing case–control risk differences for i) DUD in co-siblings of DUD probands (maroon line), ii) AUD in co-siblings of DUD probands (orange line), iii) DUD in co-siblings of AUD probands (yellow line), and iv) AUD in co-siblings of AUD probands (green line). Put another way, the maroon and green lines represent, respectively, the degree of *aggregation* in siblings of DUD and AUD. The orange and yellow lines, by contrast, represent the *co-aggregation* of, respectively, AUD in siblings of DUD probands and DUD in the siblings of AUD probands.

In figure 2a, the four lines are relatively stable for birth years 1950–1965. From birth years 1965 to 1990, by contrast, we see a striking rise in the aggregation of DUD in the siblings of DUD probands (maroon line), a less pronounced but substantial increase in the co-aggregation of DUD in siblings of AUD probands (yellow line), and a continuous moderate decline in the aggregation of AUD in the siblings of AUD probands (green line) and in the coaggregation of AUD in the siblings of DUD probands (orange line).

Figure 2b shows little difference in the pattern over birth years of the sibling aggregation of DUD and AUD and the coaggregation of AUD in the siblings of male versus female DUD probands. A modest difference, however, is seen in the pattern of the co-aggregation of DUD in the siblings of AUD probands where we have a slow monotonic rise of cumulative risk with female probands and a shallow inverted U-shaped relationship with male probands.

By contrast, we observe much more striking differences in figure 2c which depicts the pattern over birth years in the male versus female co-siblings of all probands. Consistently, across all four curves, the changes in risk between siblings of case versus control probands over birth years are greater in the male versus female co-siblings with the most dramatic differences seen in the rising risk for DUD in siblings of DUD probands. However, other effects—especially the decline in risk of AUD in siblings of AUD probands—are also more pronounced in the male co-siblings.

## Discussion

We sought, in this paper, to explore the impact on patterns of familial aggregation and coaggregation of DUD and AUD of a substantial rise in the prevalence of DUD beginning in the late 20th and extending into the early 21st century which had its greatest effect on individuals born from 1965 to 1990. We emphasize four major results. First, in the siblings of DUD versus control probands, the difference in risk for DUD *rose* dramatically beginning in birthyear 1960 while the difference in risk for AUD *declined* more slowly beginning with birthyear 1950. Second, in siblings of the AUD compared to control probands, we saw a modest rise in the risk for DUD starting at birthyear 1965 and a parallel fall in the risk of AUD. Third, we examined the patterns of results in the siblings of male versus female probands with DUD and AUD and found only modest differences. Fourth, by contrast, the results for male siblings of DUD and AUD versus control probands were considerably greater than those seen in female siblings of the DUD and AUD versus control probands.

Our results strongly suggest what might be called a *competition effect* between DUD and AUD in high-risk individual siblings. This would explain the pattern seen in figure 2a,that is, that the rise in rates of DUD in siblings of our cases versus controls DUD and AUD probands are paralleled by falls in the rates of AUD. We do not have a fully developed theoretical model to explain these results but suggest theories from behavioral economics might represent a plausible approach (Bickel, Johnson, Koffarnus, MacKillop, & Murphy, 2014). For example, Acuff et al write that ‘Behavioral economic theory suggests that behavioral allocation is sensitive to opportunity costs, or the loss of potential rewards due to the selection of another reward option (Acuff, Strickland, Aston, Gex, & Murphy, 2023) p. 157’. Rephrasing this quote into the context of the present study, it might read ‘The cost/benefit ratio may favor alcohol use under conditions in which a more desirable alternative is unavailable but may shift when an alternative, such as illicit drugs, is introduced, increasing the opportunity cost to be greater than the benefit gained from the alcohol use’. Or, to put this even more succinctly, if I have a familial/genetic predisposition to drug use disorder, behavioral economics would suggest that I am particularly incentivized to not drink and abuse alcohol as the effects are likely to be not as rewarding as that I obtain from illicit drugs if they are available.

One problem with this theory is that the risk for DUD also rises in the siblings of the AUD probands, but the rise is considerably smaller than that seen in the siblings of the DUD probands. Our hypothesis is that a proportion of the AUD cases born in the 1950s and early 1960s had little prior opportunity to use illicit drugs due to its limited availability. It would be this subgroup who, when trying illicit drugs perhaps for the first time, would find them more rewarding than alcohol and ‘switch’ their preferred drug of abuse. This hypothesis is consistent with prior results of a Swedish adoptive study of the parent-offspring transmission of genetic risk for DUD and AUD (Kendler, Abrahamsson, Ohlsson, Sundquist, & Sundquist, 2023). In that study, which examined offspring born in nearly the identical period to those examined here, a parent with AUD (by far the most common form of SUD in the parental generation) equally transmitted risk for AUD and DUD in their children. Given the relative rarity of illicit drugs in that generation, we suggested that parents with AUD likely had a relatively non-specific increased risk for SUD. By contrast, offspring of the rarer parents who had DUD had a much higher risk for DUD than for AUD—suggesting that seeking out a rarer form of SUD in the parental generation required a higher and more specific form of familial risk transmitted to their children. So, we are suggesting that a proportion of our sibling AUD probands were like the parental AUD cases from our adoption study—with a rather non-specific risk for SUD—and so some of their siblings were prone to take up DUD rather than AUD with the rising availability of illicit drugs.

We are aware of two prior studies of substance use in twins assessed over historical time periods that obtain findings broadly consistent with our own. The first examined rates of regular tobacco use in male and female twin pairs from Sweden born from 1910 to 1958 (Kendler, Thornton, & Pedersen, 2000). The rates of tobacco use and heritability of around 60% were stable in men across the entire historical period. In women, by contrast, rates of tobacco use were very low for those born in the earlier 20th century but increased dramatically to mirror those seen in men by those born in mid-century. This dramatic change was likely driven by a sharp reduction in the stigmatization of tobacco use in women. Most importantly, in women, the heritability of regular tobacco use over the same time period in women increased from nearly zero to around 60%—parallel to that seen in men.

The second study examined alcohol use in Finland in twins born from 1901 to 1957 (Virtanen, Kaprio, Viken, Rose, & Latvala, 2019). Average alcohol consumption increased over this time period in both sexes, but the changes were larger in women. Twin analyses were done in earlier and later cohorts, covering birth years 1901–1920 and 1945–1957, respectively. For women, heritability of alcohol consumption was estimated to be 21% in the earlier and 51% in the later cohort. For men, heritability in the two cohorts was estimated at 43 and 40%, respectively. Thus, we would suggest a parallel with the rise of the heritability of regular tobacco use in females in Sweden and alcohol consumption in Finland in those born over the first half of the 20th century driven by greater access to, respectively, tobacco and alcohol, and the rise in the familial aggregation of DUD in those born over the last third of the 20^th^ century resulting from the greater availability of illicit drugs.

These results have implications for the interpretation of family studies of SUD. In particular, the results of such studies will be sensitive to the rates of the form of SUD under investigation in the population at the time of the study, which in turn reflects a complex combination of the available supply of the drug, access to it for the general population, its popularity and the potential social barriers to its use. This complicates the cross-population comparison of genetic studies for SUD compared to those of other more traditional psychiatry disorders like major depression, bipolar disorder or schizophrenia where rates of illness are likely less dependent on these kinds of social, economic, and legal forces. However, our study has a further implication—that the rates of familial aggregation for one SUD in a population can be sensitive to the popularity and availability of potentially competing substances. Our study suggests a new level of complexity to the often-used term of “gene x environment interaction” when it comes to SUDs.

### Limitations

These results should be interpreted in the context of five potentially important limitations. First, our findings are dependent on the validity of the diagnoses of DUD and AUD we obtained from multiple Swedish registries. The use of registry diagnoses has a number of advantages, as neither cooperation effects, social desirability biases nor recall problems are of relevance. However, our results, which require individuals with substance use problems to have contact with medical and or legal authorities, will not replicate findings from personal interviews. The validity of our SUD diagnoses is supported by the high rates of concordance across ascertainment methods (Kendler, Lönn, Salvatore, Sundquist, & Sundquist, 2018; Kendler et al., 2015) and patterns of resemblance in relatives similar to those found in personally interviewed samples (Prescott & Kendler, 1999; Tsuang et al., 1996). We assessed the representativeness of our sibling sample in another way—comparing our patterns of familial aggregation to a more typical family study design. Calculating relative risks from our morbid risk results, we obtain a relative risk of 3.6 for DUD and 2.5 for AUD. In the classical family study of SUD by Merikangas et al. (1998), they found reassuringly similar relative risks, in first-degree relatives, for DUD (averaged across her three categories of opiates, cocaine, and cannabis) of 3.7 and for AUD of 2.4.

Second, discriminating true cohort effects on rates of DUD and AUD versus changes in ascertainment from official registries can be challenging. A range of analyses suggest that, in many Western countries over the last several generations, the diversity, availability, and normalization of use of illicit psychoactive substances have increased (*European Drug Report: Trends and Development*, 2021; Hall & Degenhardt, 2009; Parker, Parker, Aldridge, & Measham, 1998; Sweden Drug Situation 2000, 2000; Sweden Country Drug Report 2019, 2019; Sznitman, 2008; Sznitman et al., 2013). Furthermore, there is specific evidence from Sweden of rising rates of drug availability and drug use in the 1990s and 2000s (Sznitman, 2008) continuing into the 2010s (Sweden Country Drug Report 2019, 2019) as well as for declining rates of alcohol consumption, especially spirits, after 2005 (Kraus et al., 2015). For example, a history of drug use among males presenting for military conscript examination at age 18 went from ~6% in 1987–1992 to 17% in 1999 while from 1987 to 1998, the nation-wide number of hospital discharges for drug addiction doubled (Sweden Drug Situation 2000, 2000). Rates of cannabis use among young adults rose about 50% from 2006 to 2016 (Sweden Country Drug Report 2019, 2019). Also, an age-period-cohort analysis of drug abuse hospitalizations in Sweden from 1975–2010 documented a strong period effect with higher rates of hospitalization late in our ascertainment period (Giordano et al., 2014).

However, our main analyses including primary care data which typically only became available in 2000–2005 (Sundquist, Ohlsson, Sundquist, & Kendler, 2017). Could the expansion of ascertainment in these years largely drive our findings? To address this question, we repeated the main population based and sibling analyses excluding all cases of DUD and AUD only ascertained from this source (Appendix A: Figure 1 and 2). We see only modest changes suggesting that the main patterns of our findings are unlikely to arise from changes in DUD and AUD ascertainment.

Third, we studied only siblings and so cannot disentangle the effects on familial aggregation of genetic and shared environmental factors. Indeed, for SUDs there is the added complication of contagion as we have produced evidence in Sweden that contagious processes may be at work among siblings both for DUD (Kendler, Ohlsson, Sundquist, & Sundquist, 2019) and AUD (Kendler, Ohlsson, Sundquist, & Sundquist, 2020).

Fourth, the cultural changes in Sweden after World War II and the resulting Swedish drug and temperance culture are rather unique, so our findings may not generalize to other countries.

Finally, while our model of sibling competitive effects is plausible and consistent with our results, it must be regarded as a tentative and not definitive explanation of our findings.

## Conclusions

These results point to the importance of the impact of historical trends in the availability and attitudes towards substances of abuse, reflected in changes in prevalence, on the results of family studies, and similar effects would be expected in genetic studies using molecular methods. These changes are likely to have greater effects on those with high versus low familial/genetic liability to SUDs. To further complicate this picture, our findings suggest that changes in the availability/popularity of one form of SUD can impact on the patterns of familial aggregation and genetic risk of other psychoactive substances. Finally, these findings call on the need to develop new analytic methods to incorporate these population level effects into family and genetic investigations of SUDs.

## Supporting information

Kendler et al. supplementary material Kendler et al. supplementary material

## Supplementary material

The supplementary material for this article can be found at http://doi.org/10.1017/S0033291725000327.

## Access to data and data analysis

Kristina Sundquist MD PhD had full access to all the data in the study and takes responsibility for the integrity of the data and the accuracy of the data analysis.

## Location of where work was done

Lund University, Virginia Commonwealth University.
