# Supplementary material for: The familial aggregation and co-aggregation of drug use disorder and alcohol use disorder in siblings of affected individuals born 1950–1990: A birth cohort exposed to rising rates of drug use disorder
Source: Psychol Med. 2025 Mar 5;55:e72. doi: 10.1017/S0033291725000327 (PMC12055021; doi:10.1017/S0033291725000327)

Supplementary Material

Table 1 – Description of Registers

*Total Population Register*

The Total Population Register was created digitally in 1968 and includes yearly information, from the National Tax Board, on individuals registered in Sweden. It is possible to retrieve information on e.g. birth, death, immigration, emigration, migration within Sweden, place of residence, family information, civil status, etc. For more information, see [*https://www.scb.se/contentassets/8f66bcf5abc34d0b98afa4fcbfc0e060/rtb-bar-2016-eng.pdf*](https://www.scb.se/contentassets/8f66bcf5abc34d0b98afa4fcbfc0e060/rtb-bar-2016-eng.pdf)

*Multi-Generation Register*

The Multi-Generation Register is a register made up of persons who have been registered in Sweden at some time since 1961 and those who were born in 1932 or later. These are called index persons. The register contains connections between index persons and their biological parents. In 2016, about 12 million index persons were included in the register. The Multi-Generation Register is a part of the register system for Total Population Register, where information comes from the National Tax Board. Every year, a new version of the register is created, including new index persons who immigrated or were born during the year. Information from the Multi-Generation Register may be disclosed for research and statistical purposes. See *Statistics Sweden, Background Facts, Population and Welfare Statistics 2017:2, Multi-generation register 2016. A description of contents and quality*

*National Patient Register*

In the 1960's the National Board of Health and Welfare started to collect information regarding in-patients at public hospitals, the National Patient Register (NPR). Initially it contained information about all patients treated in psychiatric care and approximately 16 percent of patients in somatic care. The register at that time covered six of the 26 county councils in Sweden. In 1984, the Ministry of Health and Welfare together with the Federation of County Councils decided a mandatory participation for all county councils. From 1987, NPR includes all in-patient care in Sweden. Since 2001, the register also covers outpatient doctor visits including day surgery and psychiatric care from both private and public caregivers. For more information, see *https://www.socialstyrelsen.se/en/statistics-and-data/registers/national-patient-register/*

*Primary Care Data*

We also used information from our new Primary Care research dataset including individual-level information on clinical diagnoses from primary health care centers from the following Swedish counties: Blekinge (2009-2018), Dalarna (2005-2018), Gotland (2011-2018), Gävleborg (2010-2018), Halland (2007-2018), Jönköping (2008-2018), Kalmar (2007-2018), Kronoberg (2006-2018), Norrbotten (2001-2018), Skåne (1989-2018), Stockholm (2003-2018), Södermanland (1992-2018), Uppsala (2005-2018), Västra Götaland (2000-2018), Värmland (2005-2018), Västerbotten (1991-2018), Västernorrland (2008-2018), Västmanland (2014-2018), Östergötland (1990-2018), and Örebro (2006-2018). The retrieval of data differs due to timing of digitalization of patient records. In 2018, 99% of the Swedish population lived in these 20 counties. For more information see *Sundquist, J., Ohlsson, H., Sundquist, K. et al. Common adult psychiatric disorders in Swedish primary care where most mental health patients are treated. BMC Psychiatry 17, 235 (2017).*

*Prescribed Drug Register*

The Swedish Prescribed Drug Register started in July 2005 and includes all prescribed drugs being fetched at pharmacies, linked to personal numbers. See *https://www.socialstyrelsen.se/en/statistics-and-data/registers/national-prescribed-drug-register/*

*Cause of Death Register*

The Cause of Death Register includes all deaths occurring in Sweden from 1961 (including for Swedish citizens dying abroad) and is updated yearly. There is also a historical register between the years 1952 to 1960. For more information, see *https://www.socialstyrelsen.se/en/statistics-and-data/registers/national-cause-of-death--register/*

*Criminal and Suspicion Register*

The Swedish Criminal Register and the Swedish Suspicion Register includes individual-level information on all committed crimes from 1973 and all suspicions of crimes related to an individual from 1998. For more information, see *https://polisen.se/lagar-och-regler/behandling-av-personuppgifter/polisens-register/*

Table 2 – Definition of disorders

| Disorder | Registers Used | Definition |
| --- | --- | --- |
| Drug Use Disorder  (DUD) | Hospital Discharge Register;  Outpatient Care Register;  Primary Care Data;  Prescribed Drug Register;  Cause of Death Register;  Criminal Register;  Suspicion Register | DUD was identified in the Swedish medical and mortality registries by ICD codes (ICD8: Drug dependence (304); ICD9: Drug psychoses (292), Drug dependence (304) and Nondependent abuse of drugs of type Sedatives, Opioids or Amphetamine (305E, 305F, 305H); ICD10: Mental and behavioral disorders due to psychoactive substance use (F10-F19), except those due to alcohol (F10) or tobacco (F17)); in the Suspicion Register by codes 3070, 5010, 5011, and 5012, that reflect crimes related to DA; and in the Crime Register by references to laws covering narcotics (law 1968:64, paragraph 1, point 6) and drug-related driving offences (law 1951:649, paragraph 4, subsection 2 and paragraph 4A, subsection 2). DA was identified in individuals (excluding those suffering from cancer) in the Prescribed Drug Register who had retrieved (in average) more than four defined daily doses a day for 12 months from either of Hypnotics and Sedatives (Anatomical Therapeutic Chemical (ATC) Classification System N05C and N05BA), Opioids (ATC: N02A) or Methadone (ATC: N07BC). |
| Alcohol Use Disorder  (AUD) | Hospital Discharge Register;  Outpatient Care Register;  Primary Care Data;  Prescribed Drug Register;  Cause of Death Register;  Criminal Register;  Suspicion Register | Alcohol Use Disorder (AUD) was identified in the Swedish medical and mortality registries by ICD codes: ICD8: 571.0, 291, 303; ICD9: V79B, 305A, 357F, 571A-D, 425F, 535D, 291, 303; ICD 10: E24.4, G31.2, G62.1, G72.1, I42.6, K29.2, K70, K85.2, K86.0, O35.4, F10.1-F10.9; in the Swedish Criminal Register and the Swedish Suspicion Register with at least two registrations of drunk driving (suspicion code 3005, law 1951:649 (paragraph 4 and 4A)) or drunk in charge of a maritime vessel (suspicion code 3201, law 1994:1009 (chapter 20, paragraph 4 and 5)); in the Prescribed Drug Register by the drugs disulfiram (Anatomical Therapeutic Chemical (ATC) Classification System N07BB01), acamprosate (N07BB03), and naltrexone (N07BB04). |

Table 3 – Algorithm used to decide whether an individual with both DUD and AUD diagnoses should be counted as a case of either DUD or AUD

|  | | Number of lifetime AUD diagnoses | | | | |
| --- | --- | --- | --- | --- | --- | --- |
|  |  | 1 | 2 | 3-5 | 6-10 | >10 |
| Number of lifetime DUD diagnoses | 1 | Last diagnosis | Last diagnosis | Most common diagnosis | Most common diagnosis | Most common diagnosis |
|  | 2 | Last diagnosis | Majority of last 3 diagnoses | Majority of last 3 diagnoses | Most common diagnosis | Most common diagnosis |
|  | 3-5 | Most common diagnosis | Majority of last 3 diagnoses | Majority of last 3 diagnoses | Majority of last 3 diagnoses | Majority of last 5 diagnoses |
|  | 6-10 | Most common diagnosis | Most common diagnosis | Majority of last 3 diagnoses | Majority of last 5 diagnoses | Majority of last 5 diagnoses |
|  | >10 | Most common diagnosis | Most common diagnosis | Majority of last 5 diagnoses | Majority of last 5 diagnoses | Majority of last 5 diagnoses |

Table 4 – Details on R-packages used in statistical analyses

1. Therneau T. survival: A package for Survival Analysis in R. R package. 2024.
2. Wickham H. ggplot2: Elegant Graphics for Data Analysis. New York, NY: Springer-Verlag; 2016.
3. Wickham H, Miller E, Smith D. haven: Import and Export 'SPSS', 'Stata' and 'SAS' Files. R package. 2023.
4. Wickham H, François R, Henry L, Müller K, Vaughan D. dplyr: A Grammar of Data Manipulation. R package. 2023.
5. Barrett T, Dowle M, Srinivasan A, Gorecki J, Chirico M, Hocking T. data.table: Extension of ‘data.frame’. R package. 2024.
6. Clements M, Liu X-R, Christoffersen B. rstpm2: Smooth Survival Models, Including Generalized Survival Models. R package. 2023.
7. Neuwirth E. RColorBrewer: ColorBrewer Palettes. R package. 2022.


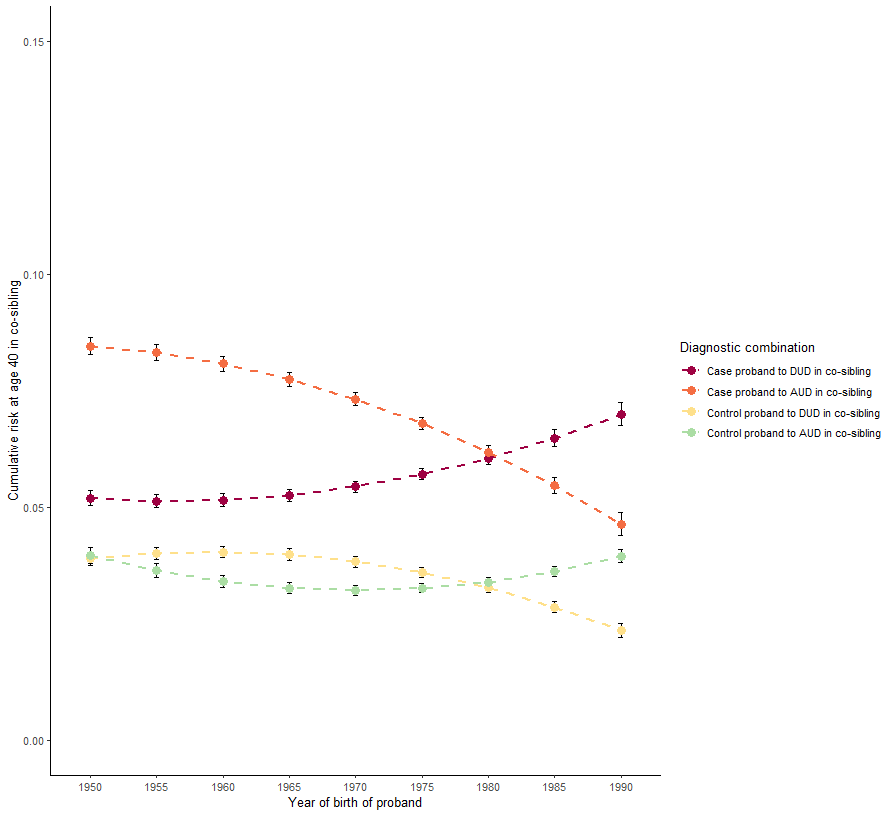

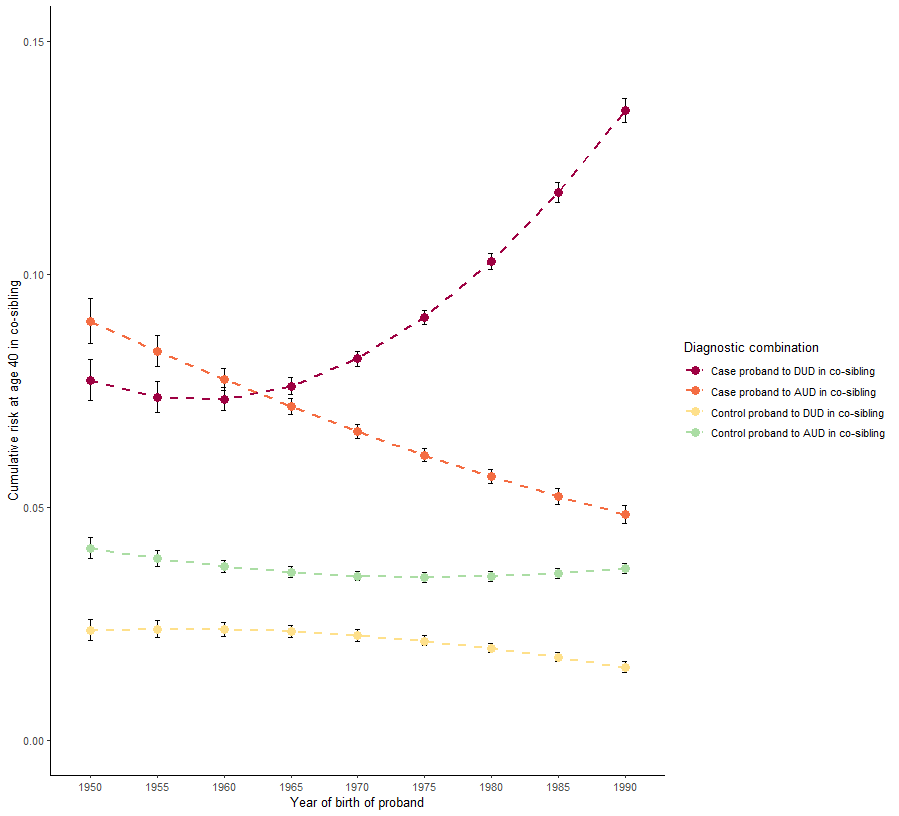
Figure 1a: Predicted mean cumulative risks (used for calculating Figure 2a) at age 40 in co-siblings. DUD probands to the left and AUD probands to the right.


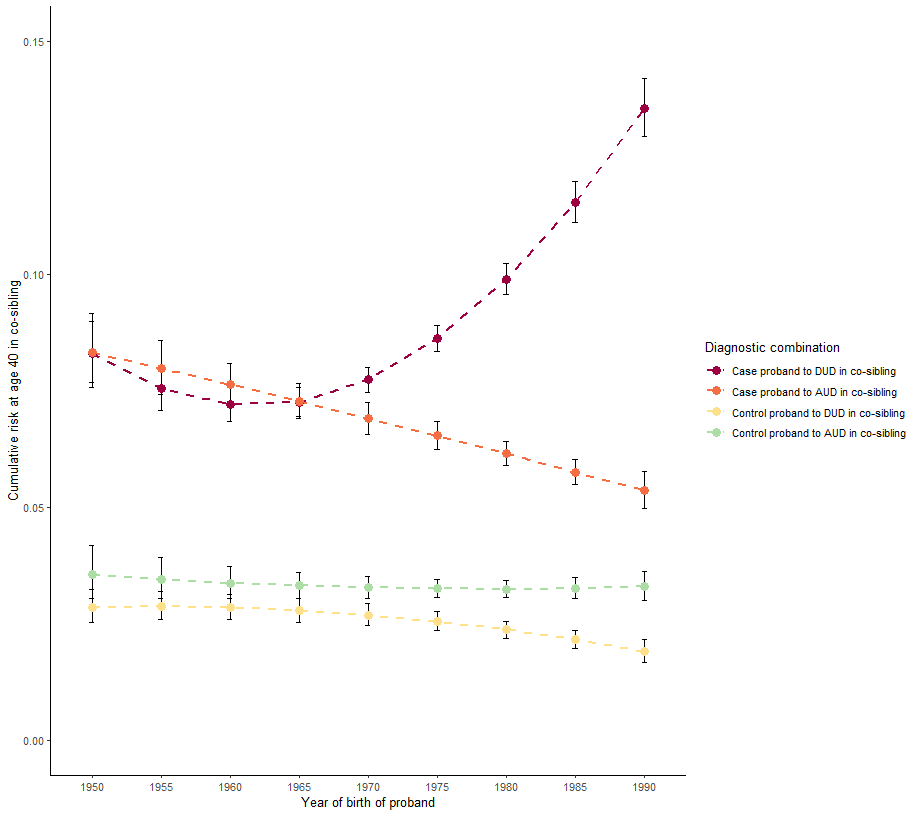

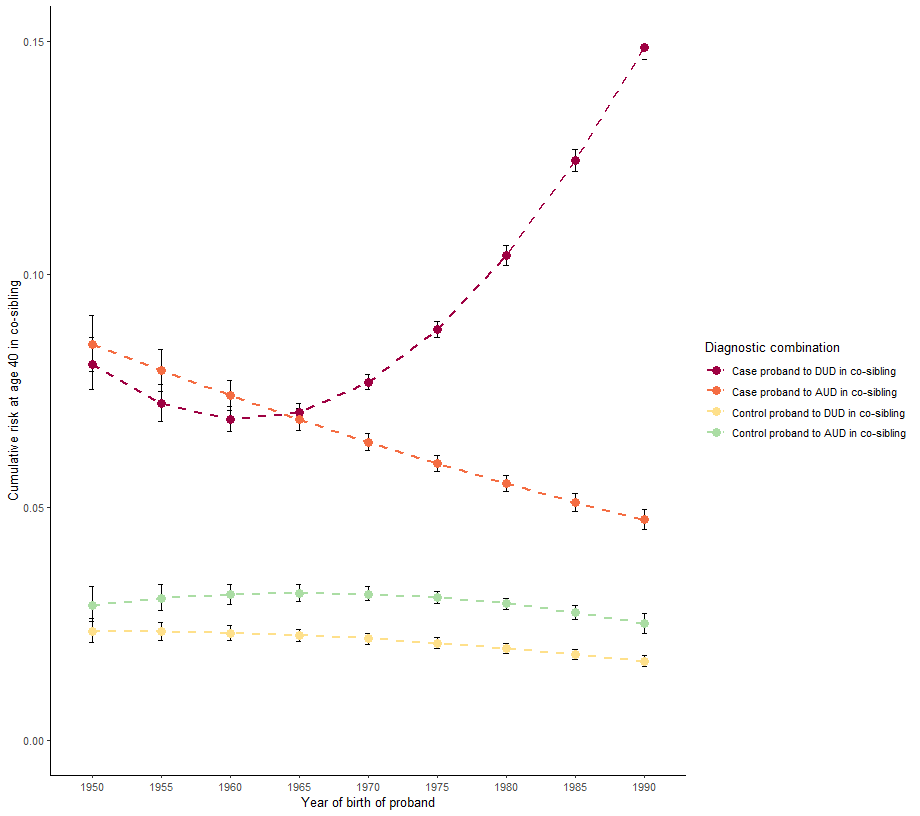
Figure 1b: Predicted mean cumulative risks (used for calculating Figure 2b) at age 40 in co-siblings. DUD probands. Figure to the left is stratified for proband sex being male, to the right female.


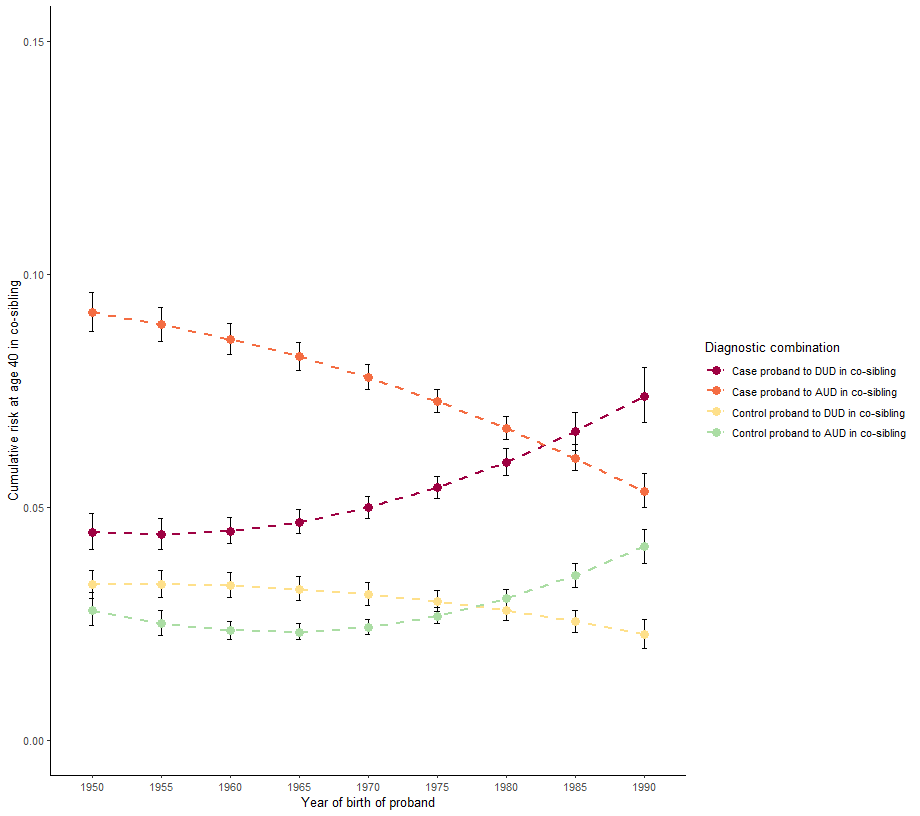

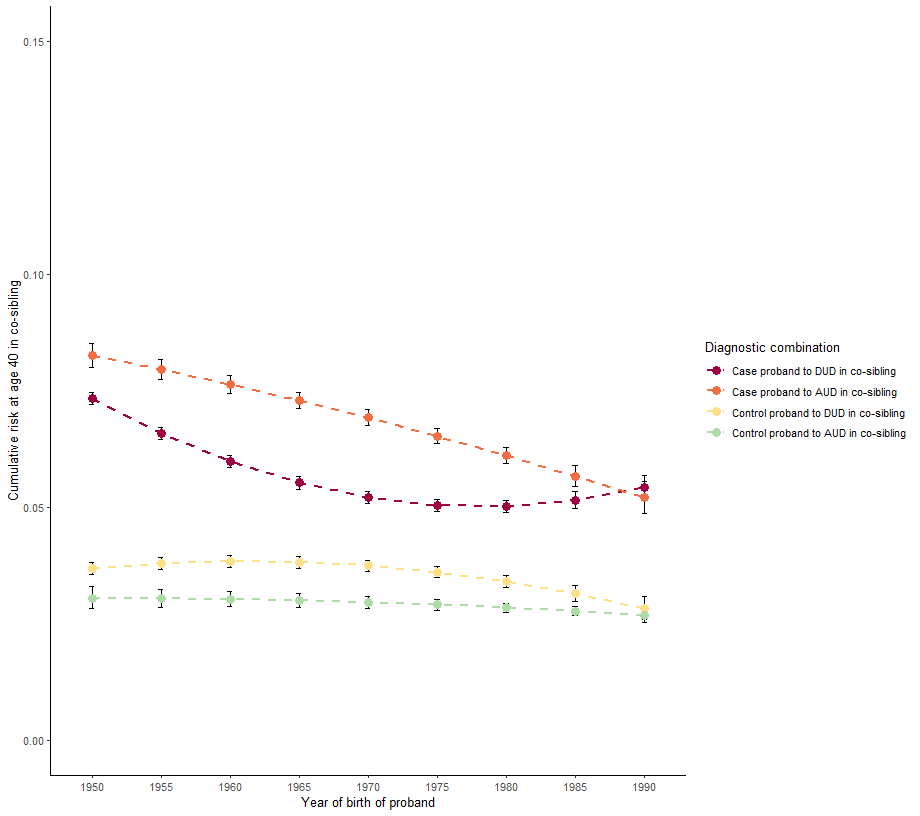
Figure 1c: Predicted mean cumulative risks (used for calculating Figure 2b) at age 40 in co-siblings. AUD probands. Figure to the left is stratified for proband sex being male, to the right female.
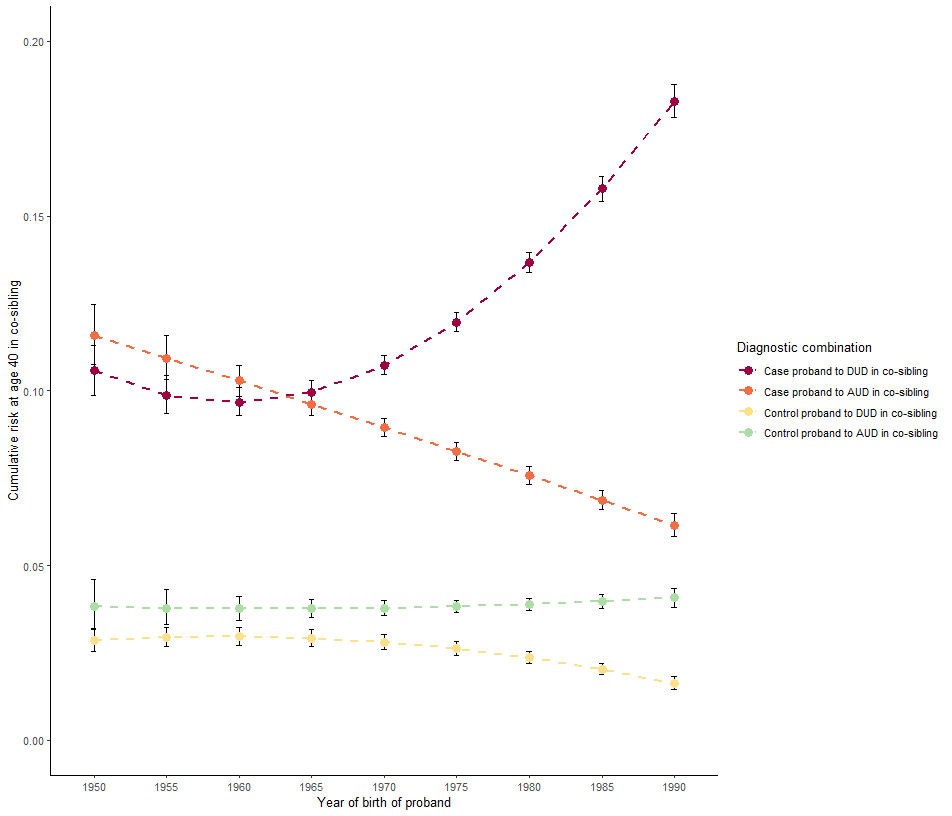
Figure 1d: Predicted mean cumulative risks (used for calculating Figure 2c) at age 40 in co-siblings. DUD probands. Figure to the left is stratified for co-sibling sex being male, to the right female.


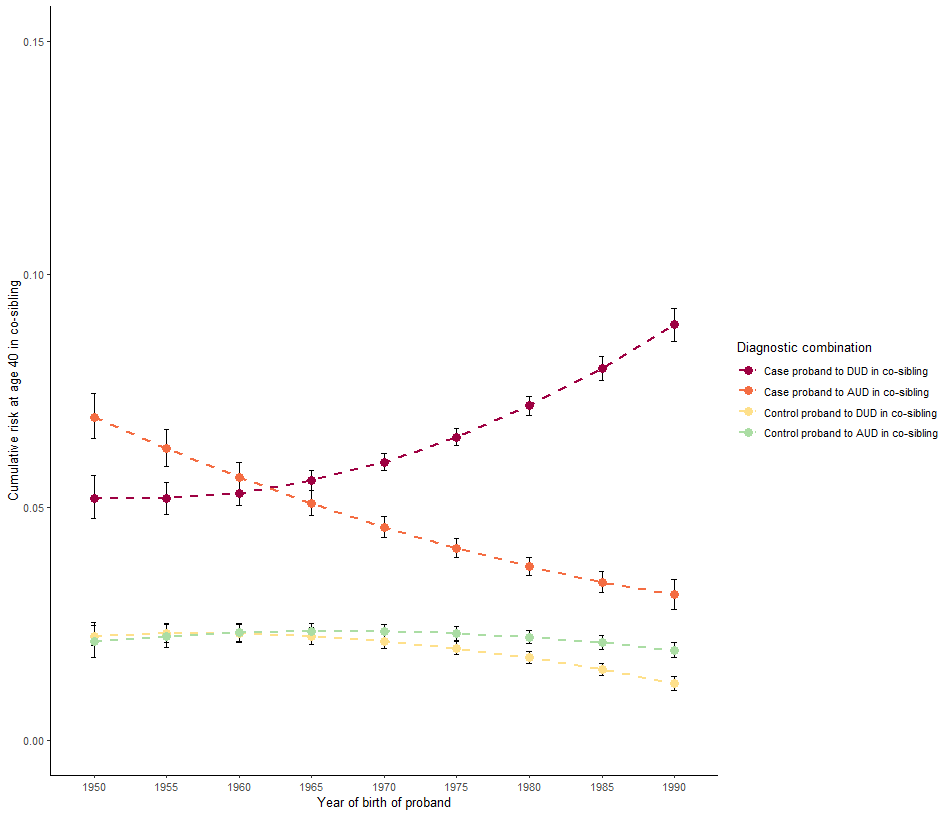


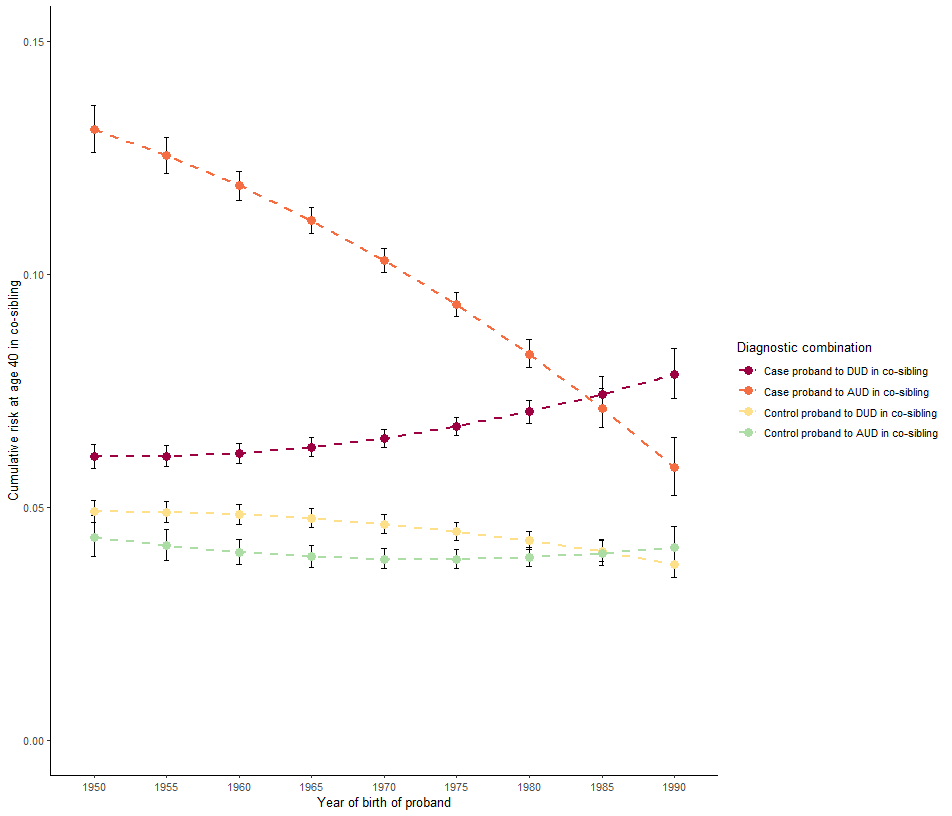
Figure 1e: Predicted mean cumulative risks (used for calculating Figure 2c) at age 40 in co-siblings. AUD probands. Figure to the left is stratified for co-sibling sex being male, to the right female.


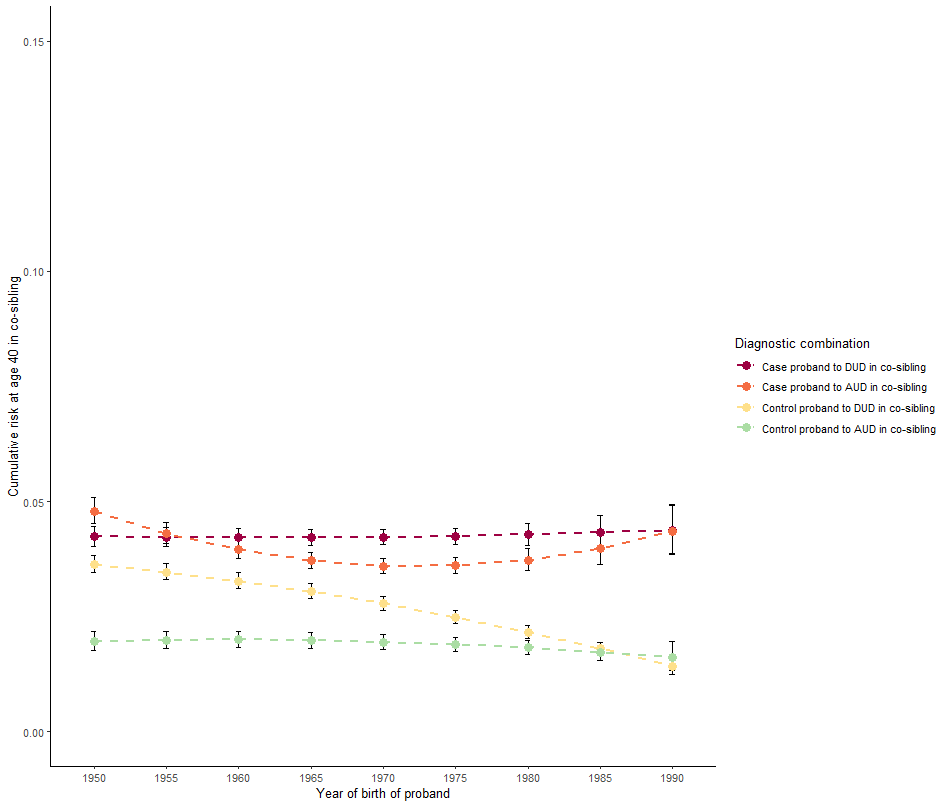


Table 5a: Regression coefficients (presented with point estimate of additive hazard per 1,000 person years (95% CI)) used to produce Figure 2a.

|  |  | Diagnostic combination | | | |
| --- | --- | --- | --- | --- | --- |
| Variable^d^ | Variable coding/transformation | Proband DUD, Co-sibling DUD^a^ | Proband DUD, Co-sibling AUD^a^ | Proband AUD, Co-sibling DUD^a^ | Proband AUD, Co-sibling AUD^a^ |
| Age, NS-K1 | Age-10 | 2.82 (1.72-3.91)^****^ | 36.65 (35.04-38.26)^****^ | 5.52 (5.04-6.00)^****^ | 65.96 (64.21-67.71)^****^ |
| Age, NS-K2 |  | 20.52 (19.03-22.02)^****^ | 59.74 (57.16-62.31)^****^ | 10.09 (9.05-11.13)^****^ | 86.55 (83.84-89.25)^****^ |
| Age, NS-K3 |  | 82.25 (78.85-85.64)^****^ | 111.60 (107.43-115.77)^****^ | 35.84 (34.06-37.62)^****^ | 134.56 (130.96-138.16)^****^ |
| Age, NS-K4 |  | -4.81 (-8.78-(-)0.85)^*^ | 180.48 (172.24-188.72)^****^ | 5.59 (3.35-7.83)^****^ | 216.79 (211.22-222.36)^****^ |
| Age, NS-K5 |  | 105.89 (99.34-112.44)^****^ | 207.13 (195.14-219.12)^****^ | 44.55 (41.33-47.77)^****^ | 227.64 (220.60-234.68)^****^ |
| Case proband | Case proband=1;  Control proband=0 | 4.66 (4.54-4.78)^****^ | 1.65 (1.50-1.79)^****^ | 0.92 (0.89-0.96)^****^ | 2.87 (2.77-2.98)^****^ |
| Year of birth proband | Year of birth-average year of birth | -0.01 (-0.01-(-)0.01)^****^ | 0.001 (-0.000-0.002) | -0.01 (-0.01-(-)0.01)^****^ | -0.006 (-0.008-(-)0.004)^****^ |
| Quadratic year of birth proband | ((Year of birth-average year of birth)^2)/10 | -0.002 (-0.003-(-)0.001)^****^ | 0.003 (0.003-0.004)^****^ | -0.006 (-0.007-(-)0.005)^****^ | 0.006 (0.005-0.007)^****^ |
| Year of birth case proband | Control proband=0;  -(Year of birth-average year of birth)/5 | -0.48 (-0.50-(-)0.45)^****^ | 0.17 (0.15-0.19)^****^ | -0.09 (-0.10-(-)0.08)^****^ | 0.11 (0.10-0.12)^****^ |
| Quadratic year of birth case proband | Control proband=0;  (-(Year of birth-average year of birth)/5)^2 | 0.06 (0.06-0.07)^****^ | -0.001 (-0.006-0.004) | 0.03 (0.03-0.03)^****^ | -0.03 (-0.04-(-)0.03)^****^ |
| Proband sex | Male=1; Female=2 | 0.02 (-0.00-0.04) | 0.04 (0.03-0.06)^****^ | 0.01 (-0.00-0.02) | 0.22 (0.20-0.23)^****^ |
| Co-sibling sex, NS-K1 | Male=1; Female=2 | 0.03 (-0.01-0.07) | -1.27 (-1.37-(-)1.17)^****^ | 0.01 (-0.02-0.04) | -1.66 (-1.72-(-)1.59)^****^ |
| Co-sibling sex, NS-K2 |  | -27.28 (-29.47-(-)25.09)^****^ | 3.36 (-0.13-6.84) | -10.85 (-12.13-(-)9.58)^****^ | 3.50 (0.87-6.12)^**^ |
| Co-sibling sex, NS-K3 |  | -21.61 (-22.86-(-)20.34)^****^ | 10.63 (7.67-13.58)^****^ | -11.56 (-12.56-(-)10.56)^****^ | -5.91 (-8.31-(-)3.51)^****^ |
| Year of birth co-sibling, NS-K1 | Year of birth-average year of birth | 0.13 (0.12-0.13)^****^ | 0.03 (0.02-0.03)^****^ | 0.04 (0.04-0.05)^****^ | 0.06 (0.06-0.07)^****^ |
| Year of birth co-sibling, NS-K2 |  | -0.66 (-0.85-(-)0.46)^****^ | -0.29 (-0.57-(-)0.00)^*^ | 0.07 (-0.07-0.22) | -1.86 (-2.12-(-)1.60)^****^ |
| Year of birth co-sibling, NS-K3 |  | 1.27 (1.11-1.44)^****^ | -0.10 (-0.37-0.16) | 0.71 (0.59-0.84)^****^ | -1.81 (-2.04-(-)1.57)^****^ |
| Quadratic year of birth co-sibling | ((Year of birth-average year of birth)^2)/10^b^ | 0.16 (0.15-0.16)^****^ | -0.01 (-0.01-(-)0.01)^****^ | 0.04 (0.04-0.04)^****^ | 0.01 (0.01-0.01)^****^ |
| Cubic year of birth co-sibling | ((Year of birth-average year of birth)^3)/100^c^ | 0.04 (0.03-0.04)^****^ | -0.005 (-0.005-(-)0.004)^****^ | 0.02 (0.01-0.02)^****^ | 0.004 (0.003-0.005)^****^ |
| Age at onset case proband, NS-K1 | 0 for control proband;  -(Age at onset)/5 | 0.26 (0.24-0.28)^****^ | 0.09 (0.06-0.11)^****^ | 0.03 (0.03-0.04)^****^ | 0.14 (0.12-0.15)^****^ |
| Age at onset case proband, NS-K2 |  | 1.21 (1.01-1.42)^****^ | -0.06 (-0.29-0.17) | -0.03 (-0.10-0.03) | 1.07 (0.91-0.24)^****^ |
| Age at onset case proband, NS-K3 |  | 1.90 (1.71-2.09)^****^ | 1.07 (0.76-1.37)^****^ | 0.77 (0.66-0.89)^****^ | 1.80 (1.56-2.03)^****^ |
| Age at onset case proband, NS-K4 |  | 0.66 (0.33-0.98)^****^ | 0.91 (0.40-1.43)^***^ | -0.15 (-0.30-0.01) | 0.87 (0.54-1.20)^***^ |
| Age at onset case proband, NS-K5 |  | 5.67 (5.27-6.06)^****^ | 1.94 (1.62-2.27)^****^ | 2.80 (2.65-2.96)^****^ | 0.46 (0.23-0.69)^****^ |
| ^a^Significance levels for p-values: *<.05, **<.01, ***<.001, ****<.0001  ^b^For Proband DUD, co-sibling DUD, transformed with division by 20 instead of 10  ^c^For Proband DUD, co-sibling DUD, transformed with division by 200 instead of 100  ^d^NS-K1 is abbreviated for natural spline, knot 1 (placement of knots chosen automatically in the stpm2 function), i.e. effect is allowed to vary over time scale attained age in co-sibling | | | | | |

Table 5b: Regression coefficients (presented with point estimate of additive hazard per 1,000 person years (95% CI)) used to produce Figure 2b proband male.

|  |  | Diagnostic combination | | | |
| --- | --- | --- | --- | --- | --- |
| Variable^b^ | Variable coding/transformation | Proband DUD, Co-sibling DUD^a^ | Proband DUD, Co-sibling AUD^a^ | Proband AUD, Co-sibling DUD^a^ | Proband AUD, Co-sibling AUD^a^ |
| Age, NS-K1 | Age-10 | 16.17 (15.09-17.25)^****^ | 38.67 (36.90-40.44)^****^ | -6.39 (-6.72-(-)6.07)^****^ | 76.16 (74.05-78.26)^****^ |
| Age, NS-K2 |  | 37.34 (35.55-39.13)^****^ | 57.45 (54.62-60.28)^****^ | 0.78 (-0.09-1.64) | 100.17 (97.07-103.27)^****^ |
| Age, NS-K3 |  | 97.41 (92.96-101.85)^****^ | 108.04 (103.18-112.90)^****^ | 22.21 (20.81-23.60)^****^ | 149.20 (145.12-153.28)^****^ |
| Age, NS-K4 |  | 45.82 (41.45-50.19)^****^ | 195.41 (185.06-205.76)^****^ | 3.55 (1.55-5.55)^***^ | 242.80 (236.54-249.06)^****^ |
| Age, NS-K5 |  | 111.24 (103.71-118.77)^****^ | 233.31 (218.01-248.61)^****^ | 31.85 (28.89-34.81)^****^ | 247.21 (239.33-255.09)^****^ |
| Case proband | Case proband=1;  Control proband=0 | 4.66 (4.52-4.81)^****^ | 1.85 (1.69-2.01)^****^ | 1.17 (1.14-1.20)^****^ | 2.72 (2.62-2.83)^****^ |
| Year of birth proband | Year of birth-average year of birth | -0.01 (-0.01-(-)0.01)^****^ | -0.001 (-0.014-0.006)^****^ | -0.003 (-0.004-(-)0.001)^****^ | -0.002 (-0.005-0.000) |
| Quadratic year of birth proband | ((Year of birth-average year of birth)^2)/10 | -0.001 (-0.002-(-)0.000)^**^ | -0.004 (-0.006-(-)0.002)^****^ | -0.004 (-0.005-(-)0.003)^****^ | -0.001 (-0.002-0.000) |
| Year of birth case proband | Control proband=0;  -(Year of birth-average year of birth)/5 | -0.65 (-0.67-(-)0.62)^****^ | 0.10 (0.08-0.13)^****^ | 0.12 (0.12-0.13)^****^ | 0.12 (0.10-0.13)^****^ |
| Quadratic year of birth case proband | Control proband=0;  (-(Year of birth-average year of birth)/5)^2 | 0.09 (0.09-0.10)^****^ | 0.01 (0.01-0.02)^****^ | 0.04 (0.03-0.04)^****^ | -0.00 (-0.01-0.00) |
| Co-sibling sex, NS-K1 | Male=1; Female=2 | -0.04 (-0.09-0.00) | -1.43 (-1.56-(-)1.31)^****^ | 0.01 (-0.02-0.03) | -1.75 (-1.83-(-)1.68)^****^ |
| Co-sibling sex, NS-K2 |  | -32.60 (-35.29-(-)29.91)^****^ | 8.67 (4.40-12.95)^****^ | -5.70 (-6.70-(-)4.70)^****^ | 4.72 (1.68-7.75)^**^ |
| Co-sibling sex, NS-K3 |  | -21.15 (-22.54-(-)19.76)^****^ | 7.29 (3.77-10.82)^****^ | -5.67 (-6.44-(-)4.90)^****^ | -10.74 (-13.56-(-)7.91)^****^ |
| Year of birth co-sibling, NS-K1 | Year of birth-average year of birth | 0.13 (0.12-0.14)^****^ | 0.05 (0.03-0.06)^****^ | 0.04 (0.03-0.04)^****^ | 0.05 (0.04-0.06)^****^ |
| Year of birth co-sibling, NS-K2 |  | -0.62 (-0.84-(-)0.40)^****^ | -0.60 (-0.93-(-)0.30)^***^ | 0.43 (0.27-0.58)^****^ | -1.61 (-1.90-(-)1.31)^****^ |
| Year of birth co-sibling, NS-K3 |  | 1.33 (1.15-1.52)^****^ | -0.56 (-0.85-(-)0.26)^***^ | 0.39 (0.27-0.52)^****^ | -1.59 (-1.85-(-)1.32)^****^ |
| Quadratic year of birth co-sibling | ((Year of birth-average year of birth)^2)/10 | 0.09 (0.08-0.09)^****^ | 0.001 (-0.001-0.004) | 0.04 (0.03-0.04)^****^ | 0.01 (0.01-0.01)^****^ |
| Cubic year of birth co-sibling | ((Year of birth-average year of birth)^3)/100 | 0.02 (0.02-0.02)^****^ | -0.002 (-0.003-(-)0.000)^**^ | 0.01 (0.01-0.02)^****^ | 0.007 (0.006-0.008)^****^ |
| Age at onset case proband, NS-K1 | 0 for control proband;  -(Age at onset)/5 | 0.22 (0.20-0.25)^****^ | 0.13 (0.10-0.16)^****^ | 0.07 (0.07-0.08)^****^ | 0.14 (0.13-0.16)^****^ |
| Age at onset case proband, NS-K2 |  | 0.01 (-0.20-0.21) | -0.17 (-0.45-0.11) | -1.20 (-1.28-1.12)^****^ | 0.48 (0.28-0.69)^****^ |
| Age at onset case proband, NS-K3 |  | 0.74 (0.50-0.98)^****^ | 0.40 (0.03-0.77)^*^ | 0.08 (-0.05-0.20) | 1.28 (1.01-1.56)^****^ |
| Age at onset case proband, NS-K4 |  | -0.13 (-0.52-0.27) | 0.71 (0.05-1.37)^*^ | -0.78 (-0.96-(-)0.60)^****^ | 0.48 (0.09-0.86)^*^ |
| Age at onset case proband, NS-K5 |  | 5.53 (5.25-5.81)^****^ | 0.29 (-0.12-0.69) | 3.10 (2.92-3.29)^****^ | -0.41 (-0.68-(-)0.13)^**^ |
| ^a^Significance levels for p-values: *<.05, **<.01, ***<.001, ****<.0001  ^b^NS-K1 is abbreviated for natural spline, knot 1 (placement of knots chosen automatically in the stpm2 function), i.e. effect is allowed to vary over time scale attained age in co-sibling | | | | | |

Table 5c: Regression coefficients (presented with point estimate of additive hazard per 1,000 person years (95% CI)) used to produce Figure 2b proband female.

|  |  | Diagnostic combination | | | |
| --- | --- | --- | --- | --- | --- |
| Variable^b^ | Variable coding/transformation | Proband DUD, Co-sibling DUD^a^ | Proband DUD, Co-sibling AUD^a^ | Proband AUD, Co-sibling DUD^a^ | Proband AUD, Co-sibling AUD^a^ |
| Age, NS-K1 | Age-10 | -2.05 (-3.62-(-)0.47)^*^ | 37.18 (34.15-40.21)^****^ | 2.37 (0.76-3.97)^**^ | 70.71 (67.45-73.96)^****^ |
| Age, NS-K2 |  | 10.22 (7.89-12.55)^****^ | 61.46 (56.81-66.12)^****^ | 7.56 (5.54-9.58)^****^ | 91.36 (86.45-96.27)^****^ |
| Age, NS-K3 |  | 65.20 (60.70-69.69)^****^ | 113.81 (106.89-120.73)^****^ | 33.54 (30.19-36.89)^****^ | 138.45 (131.80-145.10)^****^ |
| Age, NS-K4 |  | -19.53 (-25.82-(-)13.24)^****^ | 188.76 (175.95-201.57)^****^ | 5.13 (0.81-9.45)^*^ | 229.12 (218.52-239.72)^****^ |
| Age, NS-K5 |  | 92.00 (81.96-102.04)^****^ | 218.95 (201.04-236.86)^****^ | 50.04 (43.63-56.45)^****^ | 235.76 (221.89-249.63)^****^ |
| Case proband | Case proband=1;  Control proband=0 | 3.93 (3.77-4.09)^****^ | 1.82 (1.54-2.10)^****^ | 0.95 (0.82-1.07)^****^ | 3.49 (3.21-3.78)^****^ |
| Year of birth proband | Year of birth-average year of birth | -0.01 (-0.01-(-)0.01)^****^ | -0.001 (-0.006-0.004) | -0.008 (-0.010-(-)0.005)^****^ | 0.007 (0.003-0.011)^**^ |
| Quadratic year of birth proband | ((Year of birth-average year of birth)^2)/10 | -0.003 (-0.004-(-)0.001)^**^ | 0.001 (-0.001-0.004) | -0.003 (-0.005-(-)0.001)^**^ | 0.009 (0.007-0.011)^****^ |
| Year of birth case proband | Control proband=0;  -(Year of birth-average year of birth)/5 | -0.42 (-0.47-(-)0.38)^****^ | 0.13 (0.09-0.17)^****^ | -0.14 (-0.17-(-)0.12)^****^ | 0.19 (0.16-0.23)^****^ |
| Quadratic year of birth case proband | Control proband=0;  (-(Year of birth-average year of birth)/5)^2 | 0.08 (0.07-0.09)^****^ | -0.00 (-0.02-0.01) | 0.03 (0.02-0.04)^****^ | -0.03 (-0.04-(-)0.03)^****^ |
| Co-sibling sex, NS-K1 | Male=1; Female=2 | 0.03 (-0.03-0.09) | -1.26 (-1.41-(-)1.11)^****^ | 0.04 (-0.01-0.09) | -1.55 (-1.69-(-)1.42)^****^ |
| Co-sibling sex, NS-K2 |  | -20.83 (-24.12-(-)17.53)^****^ | 3.10 (-2.55-8.76) | -8.57 (-11.05-(-)6.09)^****^ | 1.57 (-3.50-6.63) |
| Co-sibling sex, NS-K3 |  | -11.03 (-13.26-(-)8.80)^****^ | 13.31 (8.42-18.21)^****^ | -7.03 (-9.07-(-)4.98)^****^ | -13.43 (-18.04-(-)8.81)^****^ |
| Year of birth co-sibling, NS-K1 | Year of birth-average year of birth | 0.11 (0.10-0.13)^****^ | 0.05 (0.03-0.07)^****^ | 0.05 (0.04-0.06)^****^ | 0.06 (0.04-0.07)^****^ |
| Year of birth co-sibling, NS-K2 |  | -0.65 (-0.97-(-)0.33)^****^ | -0.99 (-1.48-(-)0.50)^****^ | -0.52 (-0.81-(-)0.23)^***^ | -1.78 (-2.28-(-)1.27)^****^ |
| Year of birth co-sibling, NS-K3 |  | 0.94 (0.67-1.21)^****^ | -0.78 (-1.24-(-)0.32)^***^ | 0.35 (0.12-0.59)^**^ | -1.77 (-2.24-(-)1.30)^****^ |
| Quadratic year of birth co-sibling | ((Year of birth-average year of birth)^2)/10 | 0.08 (0.07-0.08)^****^ | -0.005 (-0.008-(-)0.002)^***^ | 0.05 (0.05-0.05)^****^ | 0.01 (0.01-0.01)^****^ |
| Cubic year of birth co-sibling | ((Year of birth-average year of birth)^3)/100 | 0.02 (0.02-0.02)^****^ | -0.003 (-0.005-(-)0.000)^**^ | 0.02 (0.02-0.02)^****^ | 0.005 (0.003-0.007)^****^ |
| Age at onset case proband, NS-K1 | 0 for control proband;  -(Age at onset)/5 | 0.22 (0.19-0.25)^****^ | 0.08 (0.04-0.12)^***^ | 0.02 (0.00-0.03)^*^ | 0.16 (0.13-0.20)^****^ |
| Age at onset case proband, NS-K2 |  | 2.12 (1.84-2.39)^****^ | -0.33 (-0.70-0.04) | -0.18 (-0.34-(-)0.02)^*^ | 1.04 (0.70-1.37)^****^ |
| Age at onset case proband, NS-K3 |  | 2.42 (2.12-2.73)^****^ | 0.99 (0.51-1.47)^****^ | 0.60 (0.39-0.82)^****^ | 1.76 (1.33-2.19)^****^ |
| Age at onset case proband, NS-K4 |  | 0.95 (0.43-1.47)^***^ | -0.51 (-1.23-0.22) | 0.09 (-0.21-0.40) | 1.01 (0.39-1.62)^**^ |
| Age at onset case proband, NS-K5 |  | 5.24 (4.48-6.00)^****^ | 1.53 (1.05-2.02)^****^ | 2.03 (1.75-2.31)^****^ | 0.56 (0.14-0.98)^**^ |
| ^a^Significance levels for p-values: *<.05, **<.01, ***<.001, ****<.0001  ^b^NS-K1 is abbreviated for natural spline, knot 1 (placement of knots chosen automatically in the stpm2 function), i.e. effect is allowed to vary over time scale attained age in co-sibling | | | | | |

Table 5d: Regression coefficients (presented with point estimate of additive hazard per 1,000 person years (95% CI)) used to produce Figure 2c co-sibling male.

|  |  | Diagnostic combination | | | |
| --- | --- | --- | --- | --- | --- |
| Variable^b^ | Variable coding/transformation | Proband DUD, Co-sibling DUD^a^ | Proband DUD, Co-sibling AUD^a^ | Proband AUD, Co-sibling DUD^a^ | Proband AUD, Co-sibling AUD^a^ |
| Age, NS-K1 | Age-10 | -18.19 (-19.65-(-)16.72)^****^ | 21.09 (19.35-22.83)^****^ | -6.44 (-7.20-(-)5.68)^****^ | 28.32 (26.30-30.34)^****^ |
| Age, NS-K2 |  | -4.22 (-5.69-(-)2.76)^****^ | 29.95 (27.41-32.49)^****^ | -4.37 (-5.42-(-)3.31)^****^ | 29.76 (27.02-32.50)^****^ |
| Age, NS-K3 |  | 63.62 (60.89-66.35)^****^ | 55.17 (50.94-59.40)^****^ | 26.79 (25.18-28.40)^****^ | 60.94 (57.13-64.75)^****^ |
| Age, NS-K4 |  | -54.76 (-59.37-(-)50.15)^****^ | 115.31 (105.38-125.24)^****^ | -28.64 (-31.15-(-)26.13)^****^ | 100.22 (94.02-106.42)^****^ |
| Age, NS-K5 |  | 97.38 (90.14-104.61)^****^ | 155.00 (140.12-169.88)^****^ | 47.75 (44.40-51.09)^****^ | 126.40 (118.55-134.25)^****^ |
| Case proband | Case proband=1;  Control proband=0 | 6.31 (6.11-6.51)^****^ | 2.15 (1.96-2.33)^****^ | 1.09 (1.03-1.16)^****^ | 4.93 (4.72-5.14)^****^ |
| Year of birth proband | Year of birth-average year of birth | -0.02 (-0.02-(-)0.01)^****^ | 0.004 (-0.001-0.009) | -0.007 (-0.009-(-)0.006)^****^ | -0.005 (-0.010-0.000) |
| Quadratic year of birth proband | ((Year of birth-average year of birth)^2)/10 | -0.005 (-0.006-(-)0.003)^****^ | 0.001 (-0.002-0.005) | -0.003 (-0.004-(-)0.001)^***^ | 0.003 (0.000-0.006)^*^ |
| Year of birth case proband | Control proband=0;  -(Year of birth-average year of birth)/5 | -0.71 (-0.76-(-)0.66)^****^ | 0.27 (0.24-0.30)^****^ | -0.10 (-0.12-(-)0.08)^****^ | 0.28 (0.25-0.31)^****^ |
| Quadratic year of birth case proband | Control proband=0;  (-(Year of birth-average year of birth)/5)^2 | 0.10 (0.09-0.11)^****^ | -0.005 (-0.014-0.005) | 0.02 (0.01-0.03)^****^ | -0.03 (-0.04-(-)0.01)^****^ |
| Proband sex | Male=1; Female=2 | -0.01 (-0.04-0.02) | -0.05 (-0.12-0.01) | 0.02 (-0.01-0.04) | 0.17 (0.11-0.23)^****^ |
| Year of birth co-sibling, NS-K1 | Year of birth-average year of birth | 0.16 (0.15-0.17)^****^ | 0.02 (0.01-0.04)^**^ | 0.03 (0.02-0.04)^****^ | 0.04 (0.02-0.05)^****^ |
| Year of birth co-sibling, NS-K2 |  | 0.11 (-0.15-0.36) | -0.91 (-1.38-(-)0.44)^***^ | 0.04 (-0.15-0.23) | -1.20 (-1.63-(-)0.77)^****^ |
| Year of birth co-sibling, NS-K3 |  | 1.25 (1.04-1.47)^****^ | -0.91 (-1.34-(-)0.48)^****^ | 0.58 (0.42-0.74)^****^ | -1.40 (-1.80-(-)1.00)^****^ |
| Quadratic year of birth co-sibling | ((Year of birth-average year of birth)^2)/10 | 0.13 (0.13-0.13)^****^ | -0.001 (-0.004-0.002) | 0.06 (0.06-0.06)^****^ | -0.004 (-0.009-(-)0.000)^*^ |
| Cubic year of birth co-sibling | ((Year of birth-average year of birth)^3)/100 | 0.03 (0.03-0.03)^****^ | -0.001 (-0.002-0.001) | 0.03 (0.03-0.03)^****^ | 0.001 (-0.010-0.012) |
| Age at onset case proband, NS-K1 | 0 for control proband;  -(Age at onset)/5 | 0.36 (0.33-0.40)^****^ | 0.11 (0.07-0.14)^****^ | 0.06 (0.05-0.06)^****^ | 0.26 (0.23-0.29)^****^ |
| Age at onset case proband, NS-K2 |  | 1.64 (1.30-1.97)^****^ | -1.19 (-1.57-(-)0.80)^****^ | 0.19 (0.07-0.30)^**^ | 0.48 (0.18-0.79)^**^ |
| Age at onset case proband, NS-K3 |  | 2.00 (1.73-2.26)^****^ | -0.08 (-0.60-0.43) | 0.62 (0.47-0.78)^****^ | 2.45 (2.08-2.83)^****^ |
| Age at onset case proband, NS-K4 |  | -0.27 (-0.70-0.16) | -0.81 (-1.66-0.04) | -0.22 (-0.43-(-)0.01)^*^ | 0.46 (-0.09-1.02) |
| Age at onset case proband, NS-K5 |  | 7.46 (6.91-8.02)^****^ | 2.11 (1.57-2.65)^****^ | 1.73 (1.39-2.06)^****^ | 3.26 (2.91-3.61)^****^ |
| ^a^Significance levels for p-values: *<.05, **<.01, ***<.001, ****<.0001  ^b^NS-K1 is abbreviated for natural spline, knot 1 (placement of knots chosen automatically in the stpm2 function), i.e. effect is allowed to vary over time scale attained age in co-sibling | | | | | |

Table 5e: Regression coefficients (presented with point estimate of additive hazard per 1,000 person years (95% CI)) used to produce Figure 2c co-sibling female.

|  |  | Diagnostic combination | | | |
| --- | --- | --- | --- | --- | --- |
| Variable^b^ | Variable coding/transformation | Proband DUD, Co-sibling DUD^a^ | Proband DUD, Co-sibling AUD^a^ | Proband AUD, Co-sibling DUD^a^ | Proband AUD, Co-sibling AUD^a^ |
| Age, NS-K1 | Age-10 | -1.33 (-1.91-(-)0.75)^****^ | 11.87 (10.78-12.95)^****^ | 1.58 (1.04-2.11)^****^ | 4.07 (2.75-5.38)^****^ |
| Age, NS-K2 |  | -0.71 (-1.83-0.40) | 16.45 (14.73-18.17)^****^ | 4.76 (3.81-5.72)^****^ | 11.46 (9.67-13.25)^****^ |
| Age, NS-K3 |  | 13.81 (11.85-15.77)^****^ | 39.65 (36.36-42.93)^****^ | 19.59 (18.14-21.04)^****^ | 29.54 (27.05-32.02)^****^ |
| Age, NS-K4 |  | 25.51 (20.43-30.59)^****^ | 75.93 (67.62-84.23)^****^ | 20.24 (17.71-22.76)^****^ | 46.15 (41.30-51.01)^****^ |
| Age, NS-K5 |  | 84.96 (75.99-93.93)^****^ | 102.00 (89.68-114.32)^****^ | 28.33 (24.89-31.78)^****^ | 60.30 (53.73-66.87)^****^ |
| Case proband | Case proband=1;  Control proband=0 | 2.90 (2.76-3.04)^****^ | 1.17 (1.05-1.29)^****^ | 0.77 (0.70-0.84)^****^ | 1.15 (1.05-1.25)^****^ |
| Year of birth proband | Year of birth-average year of birth | -0.01 (-0.02-(-)0.01)^****^ | -0.005 (-0.007-(-)0.003)^****^ | -0.02 (-0.02-(-)0.02)^****^ | -0.002 (-0.004-0.000) |
| Quadratic year of birth proband | ((Year of birth-average year of birth)^2)/10 | -0.003 (-0.004-(-)0.002)^****^ | -0.003 (-0.004-(-)0.001)^***^ | -0.002 (-0.003-(-)0.001)^****^ | -0.001 (-0.003-0.000) |
| Year of birth case proband | Control proband=0;  -(Year of birth-average year of birth)/5 | -0.30 (-0.33-(-)0.27)^****^ | 0.11 (0.09-0.14)^****^ | -0.09 (-0.11-(-)0.07)^****^ | 0.05 (0.03-0.07)^****^ |
| Quadratic year of birth case proband | Control proband=0;  (-(Year of birth-average year of birth)/5)^2 | 0.03 (0.03-0.04)^****^ | 0.02 (0.01-0.02)^****^ | 0.007 (0.001-0.013)^****^ | 0.02 (0.02-0.03)^****^ |
| Proband sex | Male=1; Female=2 | 0.28 (0.25-0.31)^****^ | -0.01 (-0.04-0.02) | 0.04 (0.02-0.06)^***^ | 0.11 (0.07-0.14)^****^ |
| Year of birth co-sibling, NS-K1 | Year of birth-average year of birth | 0.12 (0.11-0.13)^****^ | 0.06 (0.04-0.07)^****^ | 0.04 (0.03-0.04)^****^ | 0.05 (0.04-0.06)^****^ |
| Year of birth co-sibling, NS-K2 |  | -1.48 (-1.79-(-)1.17)^****^ | -0.73 (-1.11-(-)0.35)^***^ | 0.25 (0.05-0.46)^*^ | -0.75 (-1.13-(-)0.36)^***^ |
| Year of birth co-sibling, NS-K3 |  | 0.35 (0.11-0.59)^**^ | -0.16 (-0.52-0.20) | -0.62 (-0.80-(-)0.44)^****^ | -1.58 (-2.01-(-)1.15)^****^ |
| Quadratic year of birth co-sibling | ((Year of birth-average year of birth)^2)/10 | 0.03 (0.02-0.05)^****^ | -0.01 (-0.01-0.01)^****^ | 0.02 (0.02-0.02)^****^ | 0.006 (0.004-0.008)^****^ |
| Cubic year of birth co-sibling | ((Year of birth-average year of birth)^3)/100 | 0.003 (0.001-0.004)^****^ | -0.005 (-0.006-(-)0.004)^****^ | 0.01 (0.01-0.01)^****^ | 0.002 (0.001-0.004)^***^ |
| Age at onset case proband, NS-K1 | 0 for control proband;  -(Age at onset)/5 | 0.08 (0.06-0.11)^****^ | 0.08 (0.06-0.10)^****^ | 0.03 (0.02-0.04)^****^ | 0.005 (-0.012-0.021) |
| Age at onset case proband, NS-K2 |  | 0.42 (0.26-0.58)^****^ | -0.03 (-0.29-0.23) | 0.44 (0.32-0.56)^****^ | 2.00 (1.72-2.27)^****^ |
| Age at onset case proband, NS-K3 |  | 1.86 (1.60-2.13)^****^ | 0.65 (0.27-1.02)^***^ | 0.55 (0.39-0.72)^****^ | 2.00 (1.67-2.32)^****^ |
| Age at onset case proband, NS-K4 |  | 0.32 (-0.18-0.82) | -0.21 (-0.82-0.39) | 0.26 (0.01-0.51)^*^ | 0.67 (0.24-1.09)^**^ |
| Age at onset case proband, NS-K5 |  | 6.92 (6.50-7.34)^****^ | 0.12 (-0.27-0.51) | 0.32 (0.17-0.48)^****^ | 2.43 (2.09-2.77)^****^ |
| ^a^Significance levels for p-values: *<.05, **<.01, ***<.001, ****<.0001  ^b^NS-K1 is abbreviated for natural spline, knot 1 (placement of knots chosen automatically in the stpm2 function), i.e. effect is allowed to vary over time scale attained age in co-sibling | | | | | |


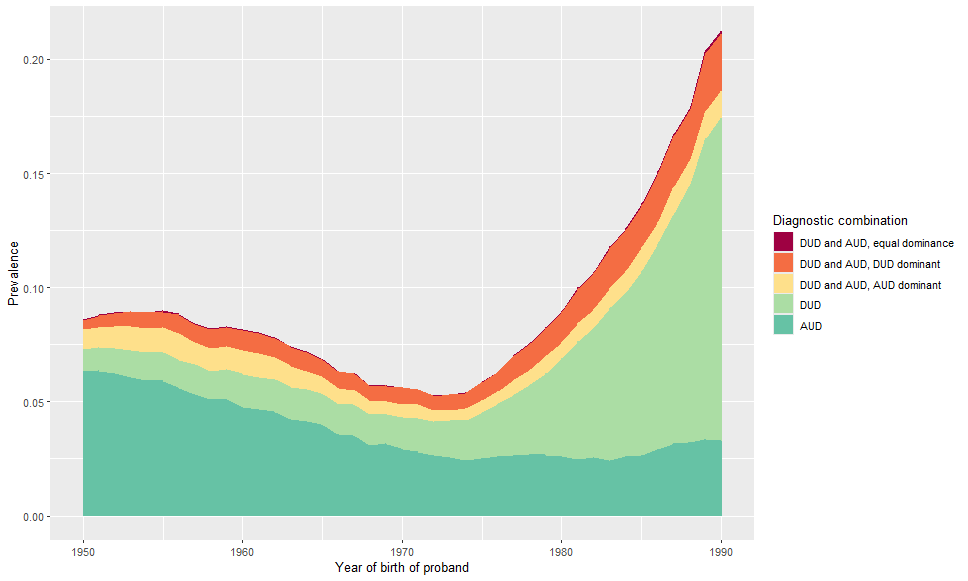
Figure 2: Lifetime prevalence in probands, with different diagnostic combinations and dominancy, born in different years. Sensitivity analysis, excluding AUD and DUD diagnoses from primary health care data.

Figure 3: Predicted mean cumulative risk (=1-survival) differences (case co-siblings – control co-siblings) at age 40 in co-siblings. Sensitivity analysis, excluding AUD and DUD diagnoses from primary health care data.


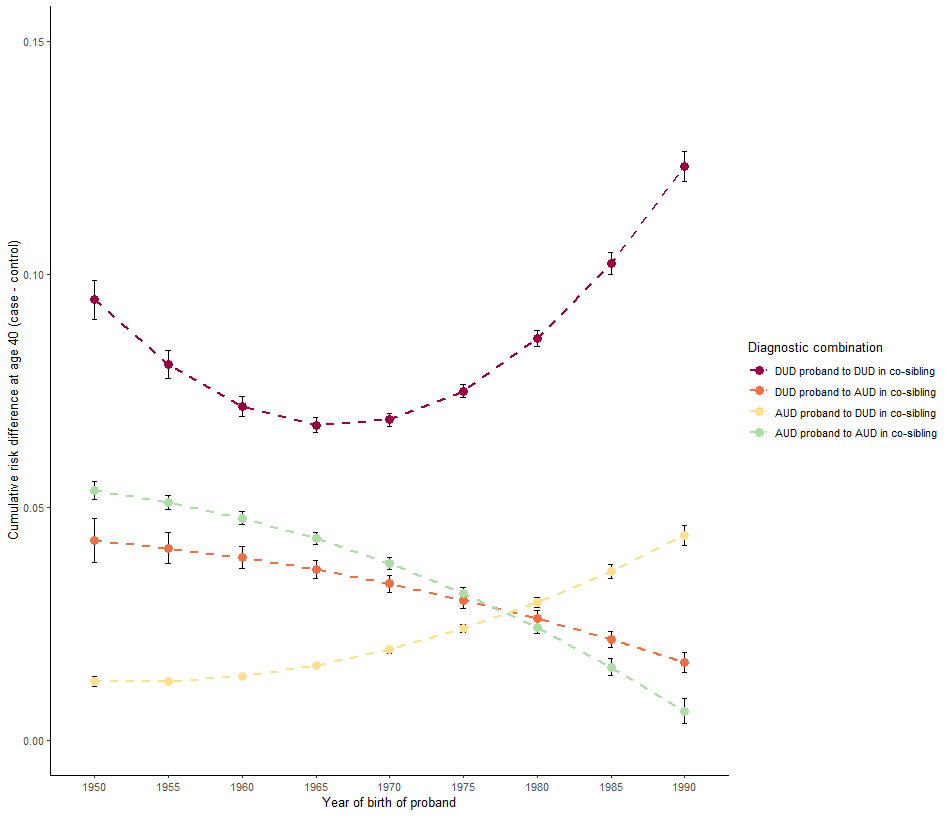


Table 6: Descriptives of total cohort, without applying hierarchy to find most dominant disorder.

|  | DUD Case Proband | DUD Control Proband | AUD Case Proband | AUD Control Proband |  |
| --- | --- | --- | --- | --- | --- |
| Number | 125,451 | 125,451 | 149,901 | 149,901 |  |
| % Female | 32.4 | 32.4 | 27.6 | 27.6 |  |
| Birth year, mean (sd) | 1976.1 (12.9) | 1976.1 (12.9) | 1967.4 (12.0) | 1967.4 (12.0) |  |
| DUD prevalence, %^a^ | 100 | 2.6 | 31.9 | 3.0 |  |
| AUD prevalence, %^a^ | 38.1 | 4.0 | 100 | 3.4 |  |
| Age at onset of DUD, mean (sd) | 30.0 (11.8) | 30.3 (11.1) | 32.1 (11.9) | 37.2 (12.7) |  |
| Age at onset of AUD, mean (sd) | 29.3 (10.9) | 29.5 (10.9) | 34.6 (13.5) | 43.8 (12.3) |  |
| Number of siblings, median (IQR) | 1 (1-2) | 1 (1-2) | 1 (1-2) | 1 (1-2) |  |
| Siblings to probands | | | | | General population |
| Number | 207,161 | 203,223 | 262,261 | 253,199 | 3,867,442 |
| % Female | 48.2 | 48.2 | 48.7 | 48.2 | 48.5 |
| Birth year, mean (sd) | 1974.5 (12.9) | 1974.8 (12.8) | 1967.0 (11.7) | 1967.3 (11.8) | 1971.6 (13.1) |
| DUD prevalence, % | 15.2 | 4.1 | 9.8 | 3.4 | 4.5 |
| AUD prevalence, % | 12.4 | 4.4 | 14.3 | 5.3 | 5.3 |
| Morbid risk of DUD, %^a^ | 21.3 | 5.8 | 11.7 | 4.1 | 5.9 |
| Morbid risk of AUD, %^a^ | 17.5 | 6.2 | 17.0 | 6.3 | 7.0 |
| Age at onset of DUD, mean (sd) | 27.8 (10.8) | 28.3 (11.1) | 32.6 (12.3) | 33.7 (12.8) | 29.9 (12.0) |
| Age at onset of AUD, mean (sd) | 30.1 (12.2) | 33.2 (13.3) | 33.8 (13.4) | 36.8 (14.0) | 34.9 (13.7) |
| Age at follow-up, mean (sd) | 43.0 (12.7) | 42.9 (12.8) | 50.1 (12.1) | 50.1 (12.1) | 45.9 (13.1) |

^a^The morbid risk represents the lifetime prevalence, with the difference that the “at risk-set” in the denominator is age-corrected so that any individual under age 18 counts 0, ages 18-44 then count 0.5 and 45 and older count 1.

Figure 4a: Predicted mean cumulative risk (=1-survival) differences (case co-siblings – control co-siblings) at age 40 in co-siblings. Without applying hierarchy to find most dominant disorder.


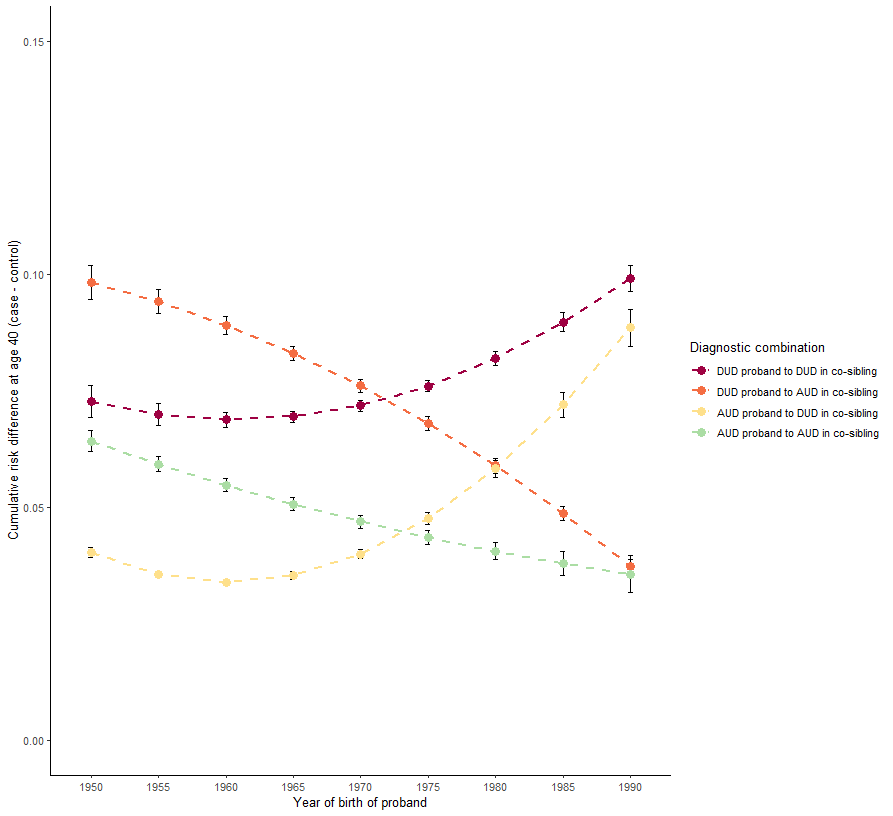


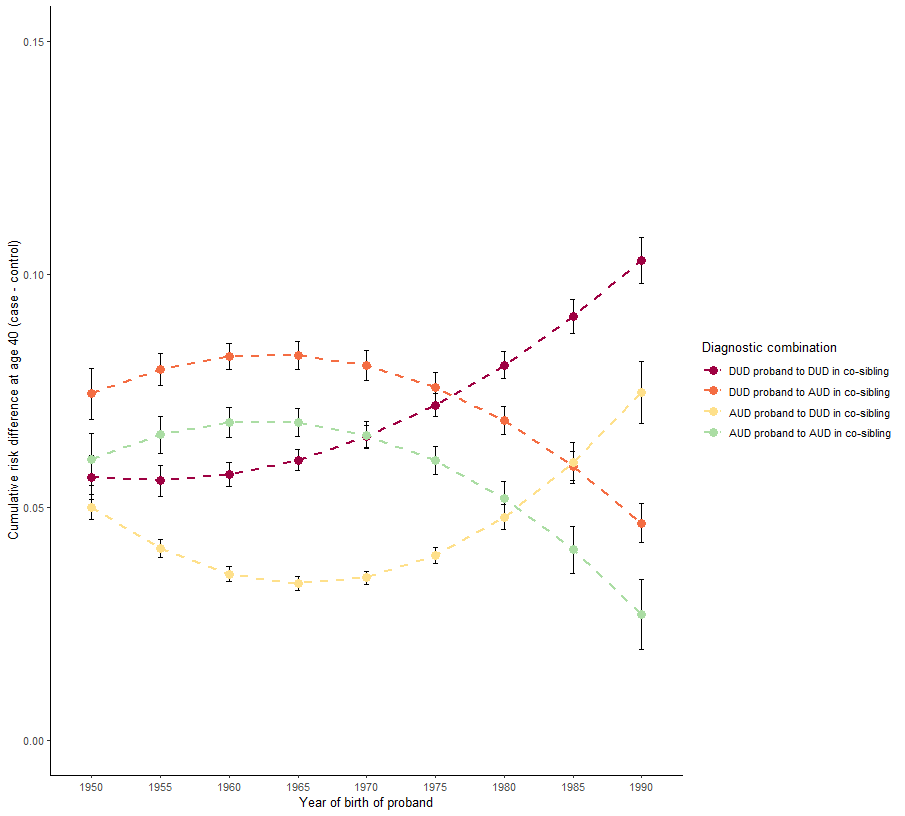
Figure 4b: Predicted mean cumulative risk (=1-survival) differences (case co-siblings – control co-siblings) at age 40 in co-siblings. Figure to the left is stratified for proband sex being male, to the right female. Without applying hierarchy to find most dominant disorder.


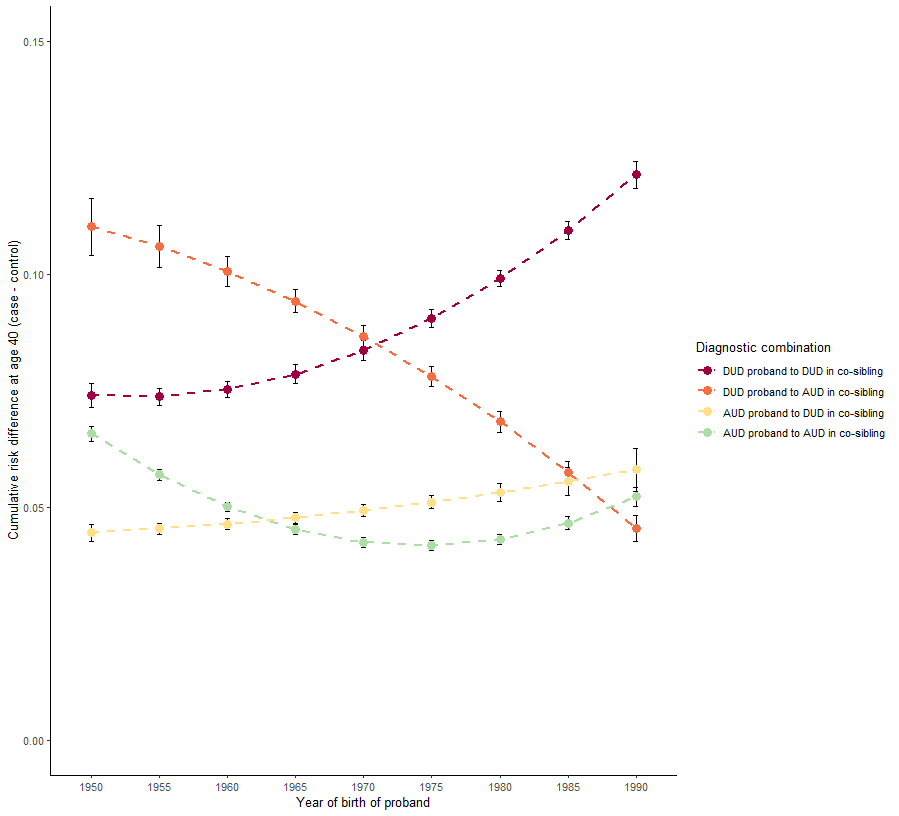


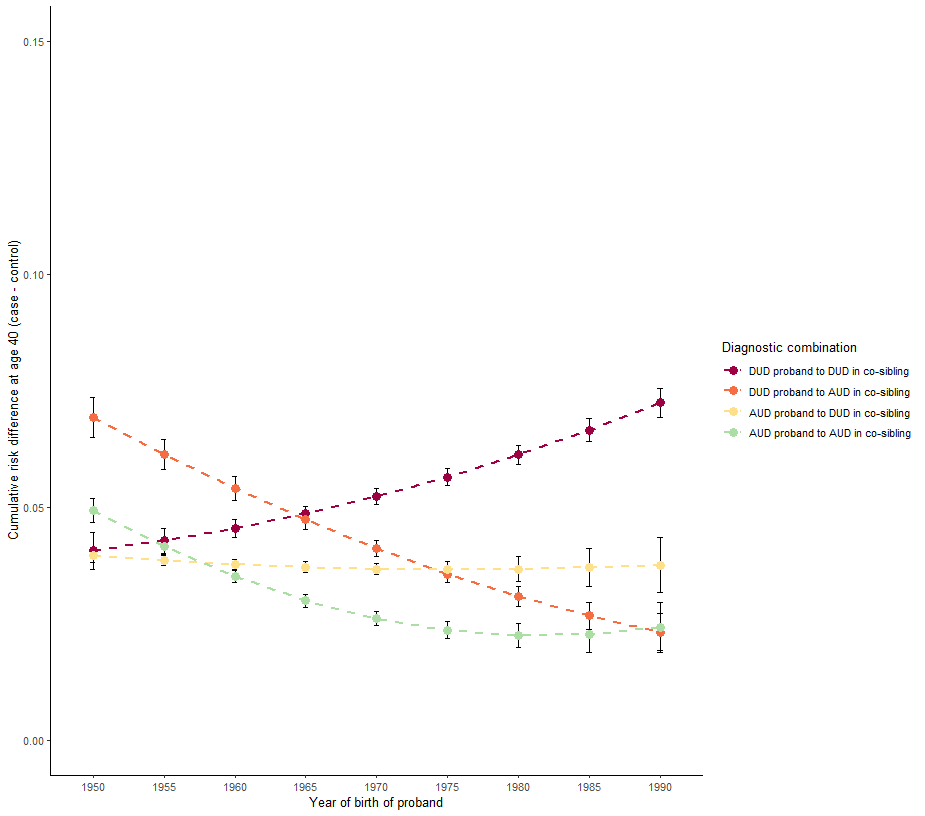

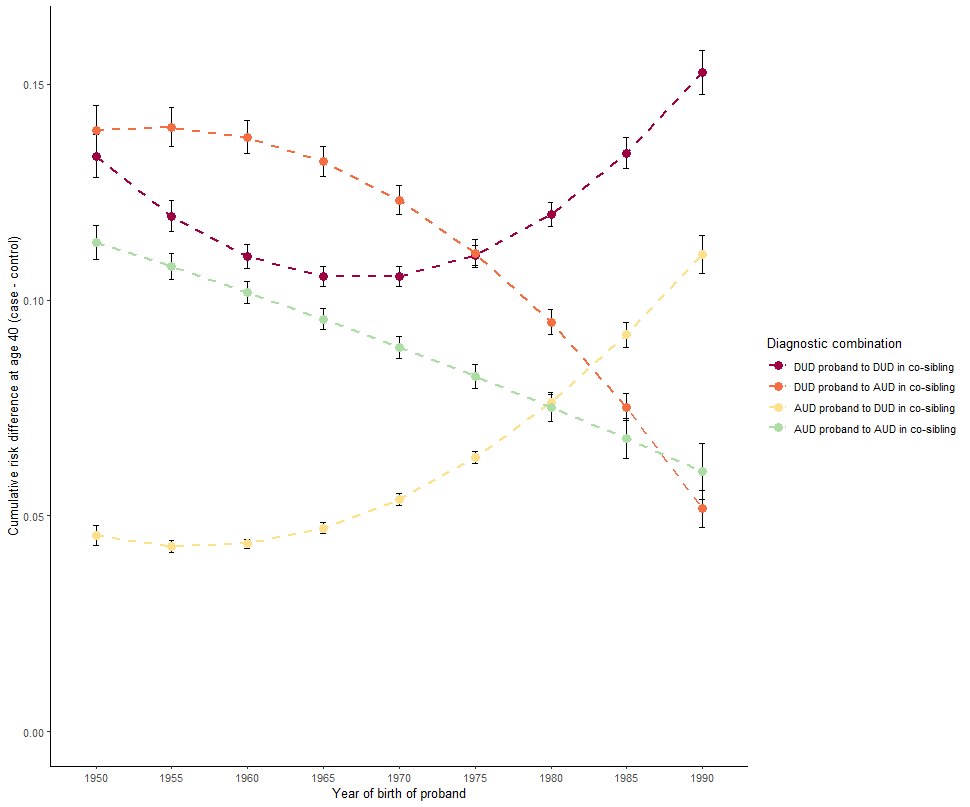
Figure 4c: Predicted mean cumulative risk (=1-survival) differences (case co-siblings – control co-siblings) at age 40 in co-siblings. Figure to the left is stratified for co-sibling sex being male, to the right female. Without applying hierarchy to find most dominant disorder.

Figure 5a: Predicted mean cumulative risks (used for calculating supplementary Figure 4a) at age 40 in co-siblings. DUD probands to the left and AUD probands to the right. Without applying hierarchy to find most dominant disorder.


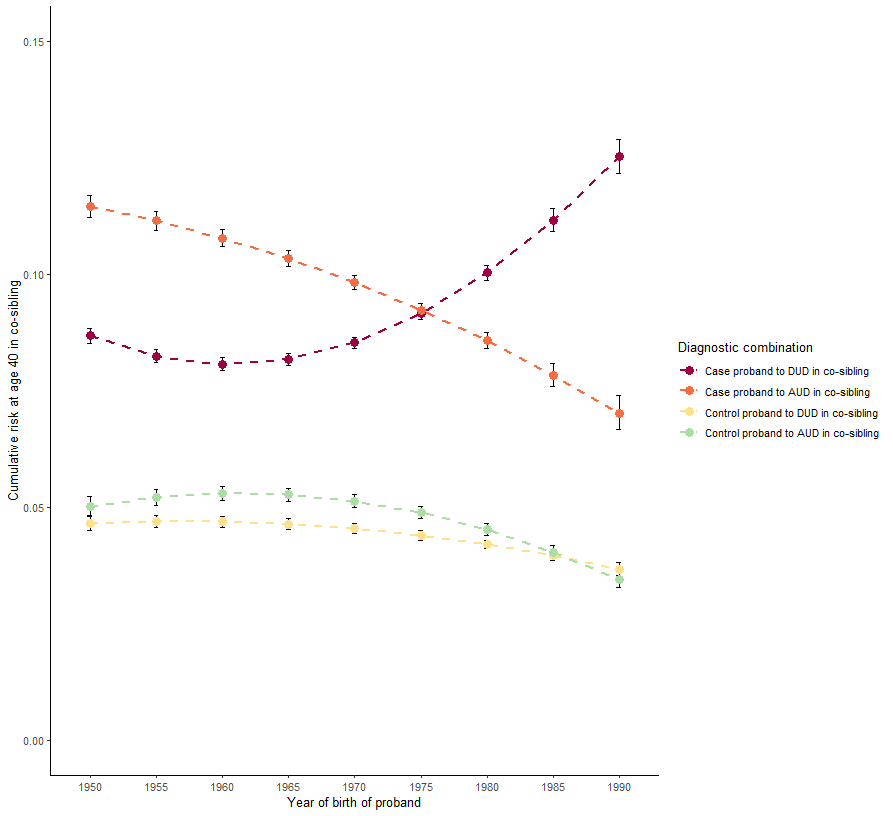

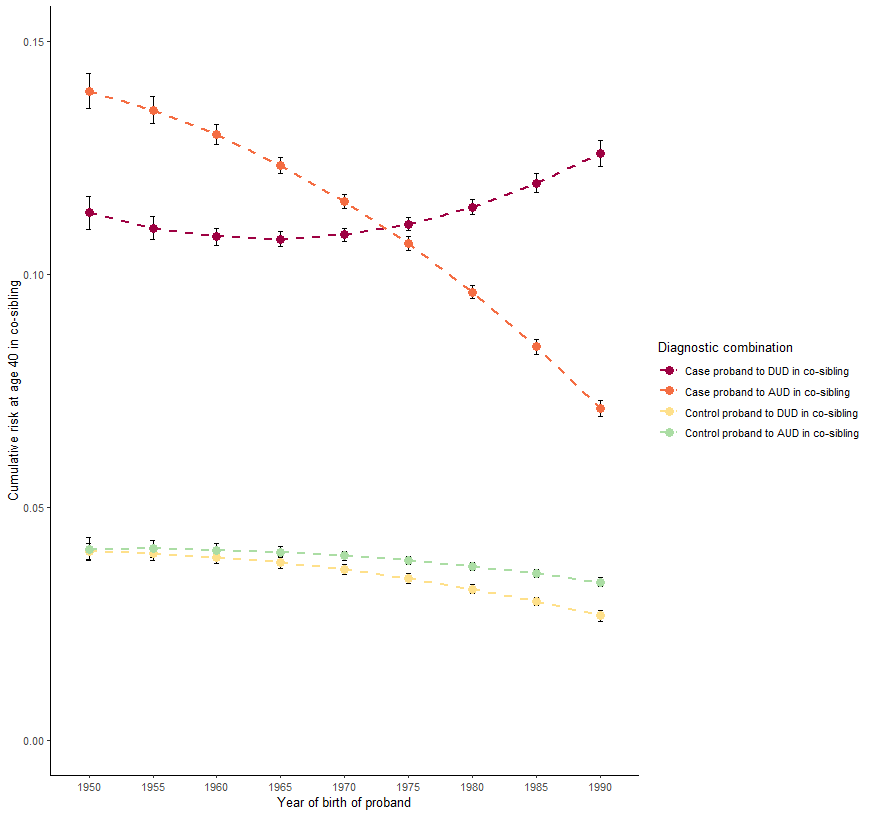


Figure 5b: Predicted mean cumulative risks (used for calculating supplementary Figure 4b) at age 40 in co-siblings. DUD probands. Figure to the left is stratified for proband sex being male, to the right female. Without applying hierarchy to find most dominant disorder.


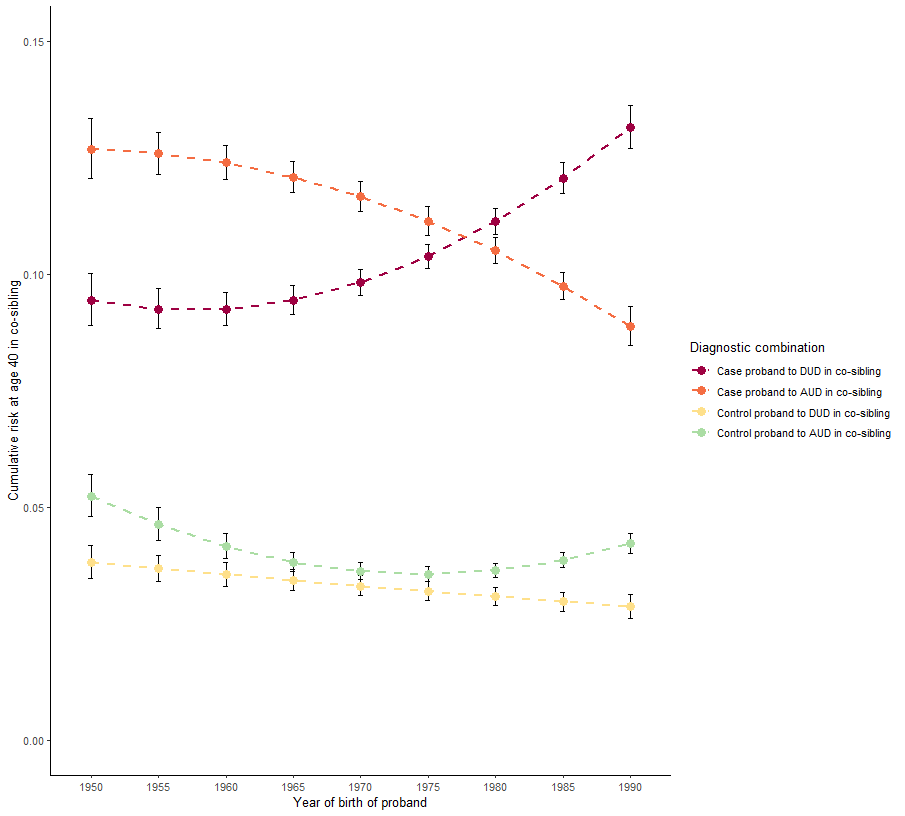

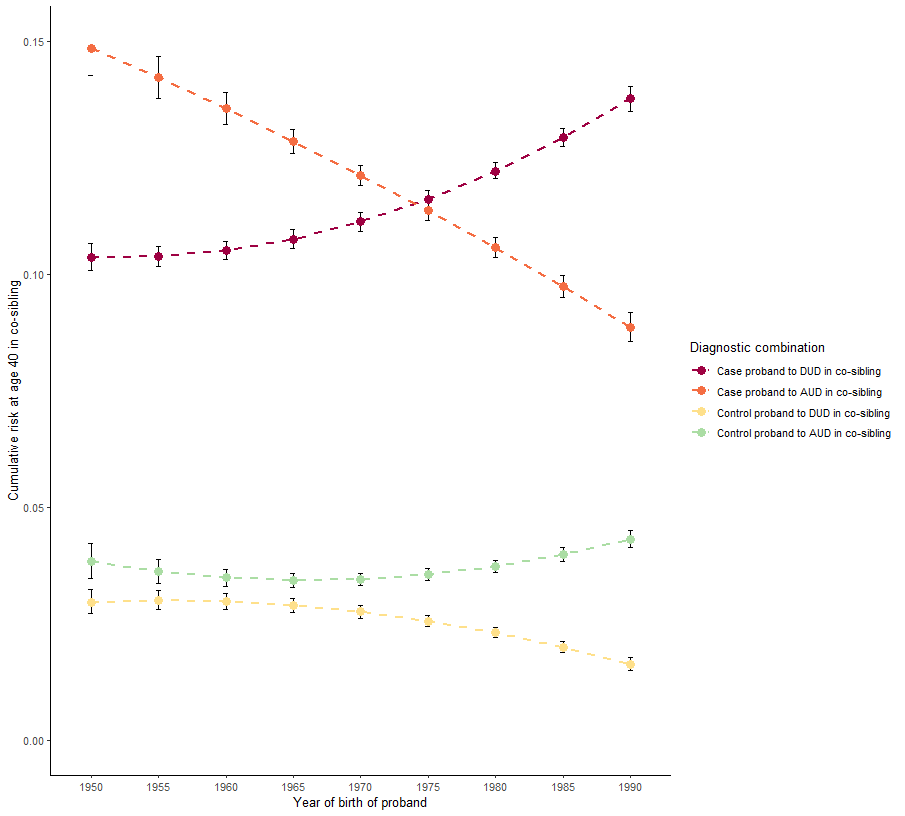


Figure 5c: Predicted mean cumulative risks (used for calculating supplementary Figure 4b) at age 40 in co-siblings. AUD probands. Figure to the left is stratified for proband sex being male, to the right female. Without applying hierarchy to find most dominant disorder.


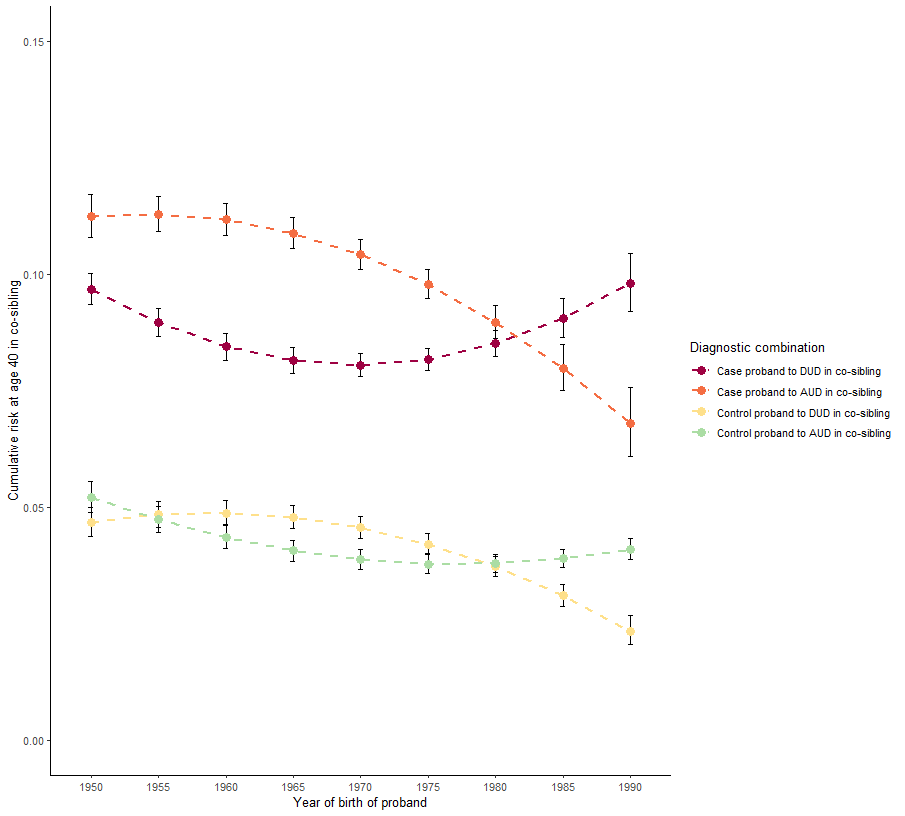

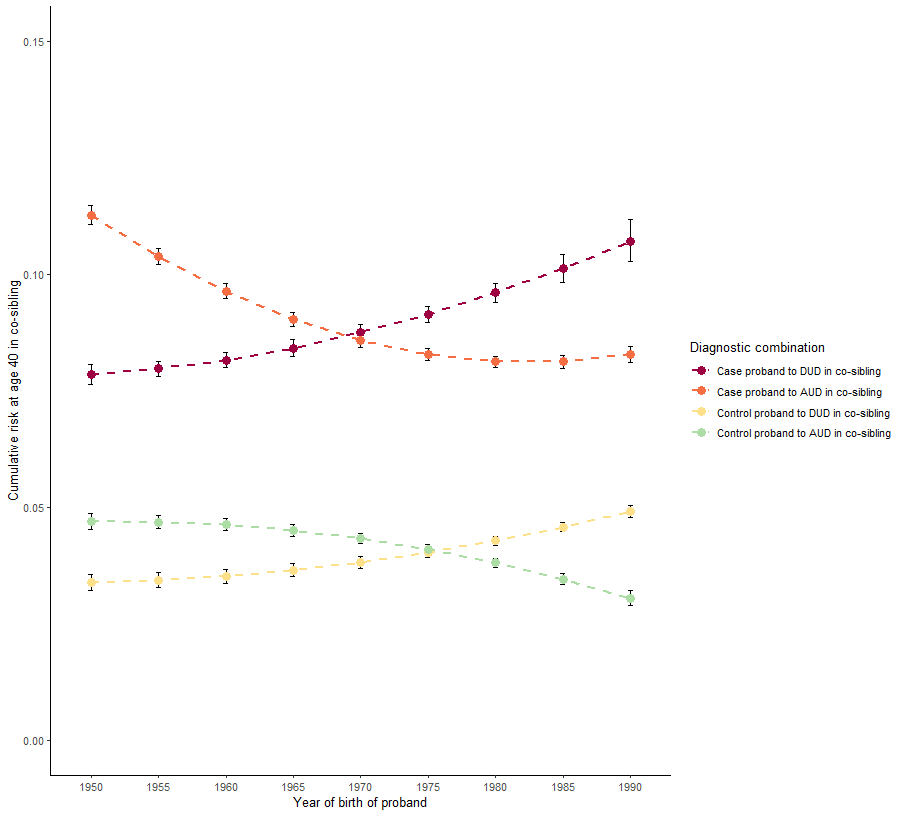


Figure 5d: Predicted mean cumulative risks (used for calculating supplementary Figure 4c) at age 40 in co-siblings. DUD probands. Figure to the left is stratified for co-sibling sex being male, to the right female. Without applying hierarchy to find most dominant disorder.


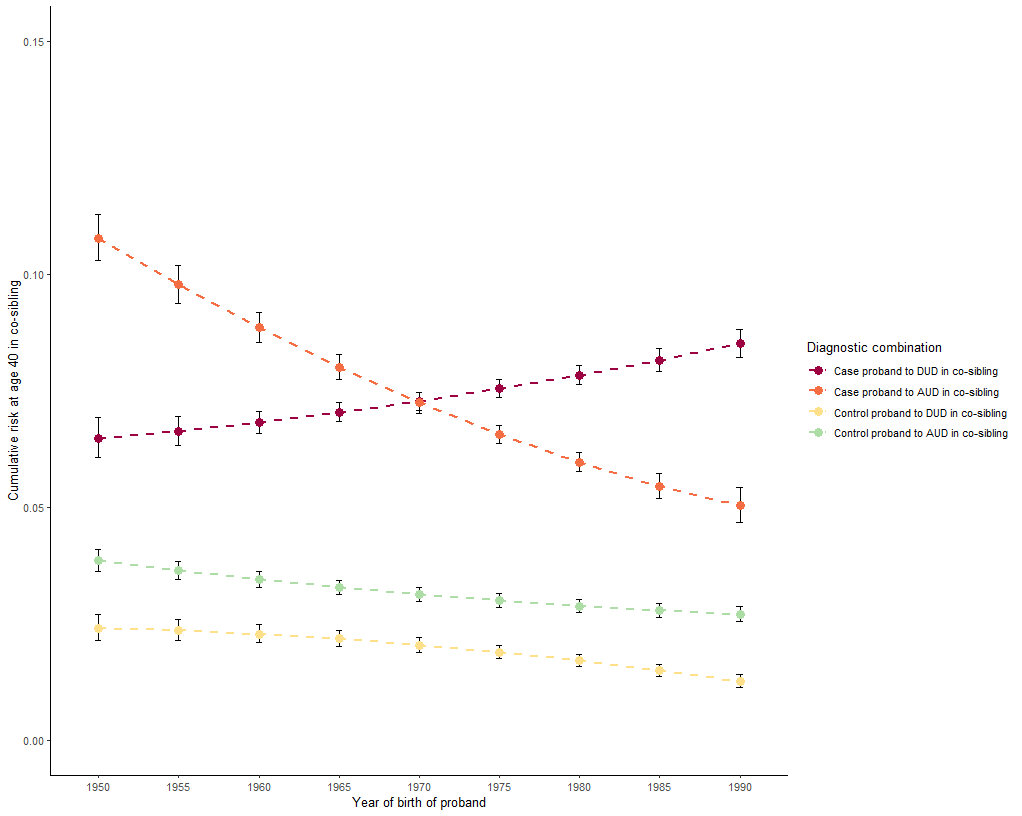

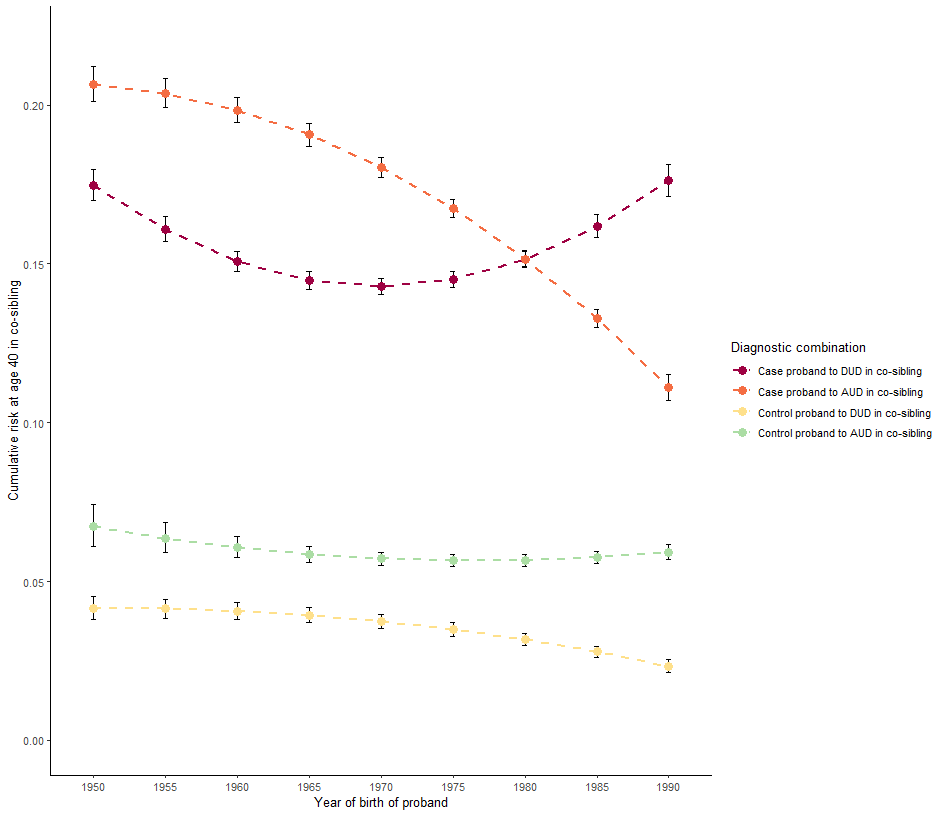


Figure 5e: Predicted mean cumulative risks (used for calculating supplementary Figure 4c) at age 40 in co-siblings. AUD probands. Figure to the left is stratified for co-sibling sex being male, to the right female. Without applying hierarchy to find most dominant disorder.


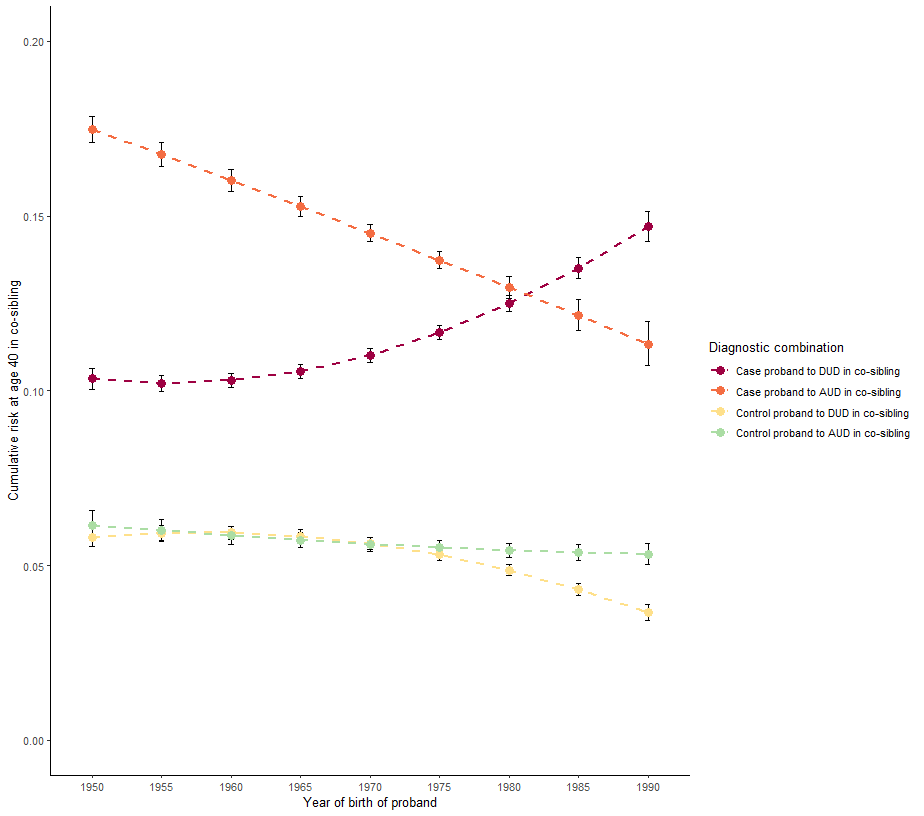

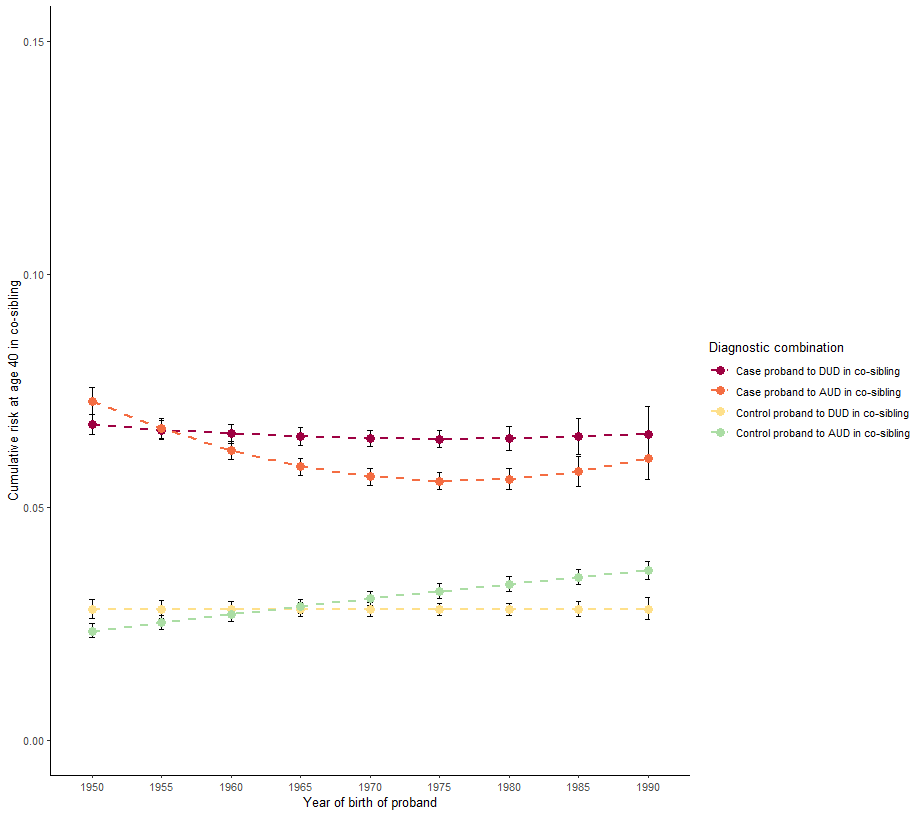

Supplement: Kendler et al. supplementary material [file S0033291725000327sup001.docx]
